# Global gene expression profiling of perirenal brown adipose tissue whitening in goat kids reveals novel genes linked to adipose remodeling

**DOI:** 10.1186/s40104-024-00994-w

**Published:** 2024-03-14

**Authors:** Le Zhao, Haili Yang, Minhao Li, Min Xiao, Xingchun Li, Lei Cheng, Wenqiang Cheng, Meixi Chen, Yongju Zhao

**Affiliations:** College of Animal Science and Technology, Southwest University, Chongqing Key Laboratory of Herbivore Science, Chongqing, 400715 China

**Keywords:** Adipose remodeling, Brown adipose tissue, Goat kids, Key thermogenesis-related genes, Whitening

## Abstract

**Background:**

Brown adipose tissue (BAT) is known to be capable of non-shivering thermogenesis under cold stimulation, which is related to the mortality of animals. In the previous study, we observed that goat BAT is mainly located around the kidney at birth, and changes to white adipose tissue (WAT) in the perirenal adipose tissue of goats within one month after birth. However, the regulatory factors underlying this change is remain unclear. In this study, we systematically studied the perirenal adipose tissue of goat kids in histological, cytological, and accompanying molecular level changes from 0 to 28 d after birth.

**Results:**

Our study found a higher mortality rate in winter-born goat kids, with goat birthing data statistics. Then we used thermal imaging revealing high temperature in goat hips at postnatal 0 d and gradually decrease during 28 d. This is consistent with the region of perirenal BAT deposition and highlights its critical role in energy expenditure and body temperature regulation in goat kids. Additionally, we found a series of changes of BAT during the first 28 d after birth, such as whitening, larger lipid droplets, decreased mitochondrial numbers, and down-regulation of key thermogenesis-related genes (*UCP1*, *DIO2*, *UCP2*, *CIDEA*, *PPARGC1a*, *C*/*EBPb*, and *C/EBPa*). Then, we used RNA-seq found specific marker genes for goat adipose tissue and identified 12 new marker genes for BAT and 10 new marker genes for WAT of goats. Furthermore, 12 candidate genes were found to potentially regulate goat BAT thermogenesis. The mechanism of the change of this biological phenomenon does not involve a large-scale death of brown adipocytes and subsequent proliferation of white adipocytes. While apoptosis may play a limited role, it is largely not critical in this transition process.

**Conclusions:**

We concluded that perirenal BAT plays a crucial role in thermoregulation in newborn goat kids, with notable species differences in the expression of adipose tissue marker genes, and we highlighted some potential marker genes for goat BAT and WAT. Additionally, the change from BAT to WAT does not involve a large-scale death of brown adipocytes and subsequent proliferation of white adipocytes.

**Supplementary Information:**

The online version contains supplementary material available at 10.1186/s40104-024-00994-w.

## Background

Dazu black goat, a local breed, is reproductively prolific, generally producing multiple kids. A high lambing rate, healthy kids, and a high survival rate are key measures that improve the goat reproductive rate. Our laboratory has analyzed the regulatory mechanisms of Dazu black goat reproduction from the perspectives of the ovary [1], testis [2], embryo implantation [3], and placental development [4]. However, prior research has revealed that kid mortality rates in Chongqing during wet and cold winters [5] are also substantially higher, causing a significant impact on the profitability of the Dazu black goat industry. How to reduce goat kid mortality is a critical issue for goat production.

Mammalian adipose tissue is an important endocrine organ, and it plays a central role in regulating whole-body energy metabolism. Adipose tissue is usually formed in a specific location at a specific time to meet the needs of organism development [6]. Adipose tissue is divided into white adipose tissue (WAT) and brown adipose tissue (BAT) [6, 7]. The WAT is often linked to energy storage. BAT expresses tissue-specific uncoupling protein 1 (UCP1) at high levels, which converts chemical energy (from lipid and glucose) into heat energy (in the form of non-shivering thermogenesis), and its content is significantly associated with mortality in young animals [8, 9]. In most mammals, including humans, goats, sheep, and mice BAT is primarily present in infancy and predominantly located in specific regions of the body such as the neck, pericardia, and perirenal regions [6, 8, 10–12]. Adipose tissue undergoes dynamic transformations across species: WAT-to-BAT in Syrian hamsters [13]. BAT-to-WAT transition in newborn rabbits [14, 15], goats [11, 16], and sheep [10]. Given BAT’s ability to generate heat, studying its thermogenesis mechanism and BAT-to-WAT transition can enhance animals’ adaptability to a huge change of environmental, which is critical for improving their survival rate, particularly among young animals. Although perirenal adipose tissue in goats undergoes a change from BAT to WAT during the postnatal period [11, 17], the same transition is not observed in mouse models [18]. While several studies have reported that goat perirenal BAT was changing to WAT after birth, and they have not elucidated the responsible mechanisms for this species-specific change in goats [11, 19]. Additionally, there is currently no systematic description of adipose tissue marker genes in goats. In present study, we systematically studied the whitening changes of goats perirenal adipose tissue in imageology, histology, cytology, and gene expression during first month which is the most important for their survive.

## Materials and methods

### Pre-weaning kid mortality statistics

All animal experiments followed the Southwest University Institutional Animal Care and Use Committee (IACUC-20221122-01) regulations. Statistics on pre-weaning mortality of Dazu black goats from 2011 to 2018 refer to previously published articles [5]. All experiments were carried out at the Dazu black goat farm in Southwest University, Chongqing, China. It is situated at an altitude of 260 m above mean sea level, with latitude and longitude positions being 29°48’44” N and 106°24’52” E, respectively.

### Animal and sample collection

The national livestock and poultry genetic resource Dazu black goat was used as the research object. All samples were collected in the winter. All the goats were kept in a semi-intensive system, allowing them to move freely in a spacious concrete barn, and given them the same management conditions such as food, water and breeding conditions. We collected data including its body weight, body size (body length, body height, chest width, and chest girth), and rectal temperature weekly from 0 to 28 d (D0, D7, D14, D21, and D28) after birth. Finally, we collected four number of goat kids in each group except D14 and D21 group with three number. Then all the healthy male goat kids were humanely euthanized and collected their all perirenal adipose tissue after body size measurement for following research. One portion of the perirenal adipose tissue was fixed with fixation solution (Servicebio, Wuhan, China) for subsequent experiments. A second portion of perirenal adipose tissue was immediately frozen in liquid nitrogen and subsequently stored at −80 °C.

### Thermographic evaluation of goat body surface temperatures

Representative infrared images of the left anatomical region (shoulder, rips, flank, lateral rump), head region (right ocular globe and forehead) and dorsal region (scapula, midloin, hips, rump) of goats were measured using a thermal imaging camera (FLIR ONE Pro; FLIR Systems, Wilsonville, OR, USA) and analyzed by FLIR Tools software (Additional file 1: Fig. S1). In this case, the thermographic camera was positioned perpendicular to the anatomical area of interest at a fixation distance of 0.5 m to the head, 1.0 m to the dorsal and left lateral regions of the goats, as previous described [20]. Thermographic images of 30 goats (D0 (*n* = 6), D7 (*n* = 6), D14 (*n* = 6), D21 (*n* = 6) and D28 (*n* = 6)) were recorded in this study and the maximum, minimum and mean temperatures (°C) in each region were analyzed.

### Hematoxylin-eosin (H&E) staining

Perirenal adipose tissue was processed by fixation with 4% neutral buffered paraformaldehyde, gradient dehydration, clearing, paraffin wax infiltration, and embedding according to standard procedures [4]. The embedded tissue was immobilized on a microtome, and serial thin sections (thickness, 5 μm) were cut. The sections were then stained with hematoxylin and eosin, dehydrated, sealed with neutral gum, and dried at room temperature for observation and photography.

### Triglyceride assay

A triglyceride (TG) assay kit (Nanjing Jiancheng Bioengineering, Nanjing, China) was used to measure the triglyceride contents of perirenal adipose tissue at D0, D7, D14, D21, and D28. All detection steps were carried out according to the manufacturer’s instructions [21].

### Immunohistochemistry

Tissue blocks immersed in 4% paraformaldehyde were retrieved, dehydrated in a gradient series, embedded in paraffin, and sectioned. Next, the sections were blocked and incubated with the primary antibody. The primary antibody UCP1 was purchased from Proteintech Group (Wuhan, China). After washing three times, the sections were incubated with a secondary antibody. The sections were then washed three times, counterstained, dehydrated, cleared, and mounted. Finally, the sections were viewed and photographed under an inverted microscope.

### Transmission electron microscopy

The morphology and number of mitochondria in adipose tissue were observed using a transmission electron microscope (TEM, Hitachi, Japan). Briefly, the perirenal adipose tissues were pre-cooled at 4 °C, and then treated with electron microscopy fixative. Using standard procedures, the samples were then dehydrated, embedded, and sliced with an ultramicrotome Leica EM UC7. The tissues were retrieved and transferred to 150 meshes cuprum grids with formvar film. The samples were then stained with a 2% uranium acetate saturated alcohol solution for 8 min (in the dark), rinsed in 70% ethanol three times, and then rinsed in ultra-pure water three times. Next, the samples were stained with 2.6% lead citrate for 8 min (in the absence of CO_2_), and rinsed with ultra-pure water three times. After removing excess liquid with filter paper, the cuprum grids were placed in a grids board and dried overnight at room temperature. Finally, the samples were observed under TEM, and images were collected for analysis.

### Quantification of relative mitochondrial DNA (mtDNA) copy numbers

DNA was extracted from the tissues using the high-salt method [22]. A DNA lysis buffer was prepared with 2.5 mL of 10% SDS, 1.1 mL of 4.5 mol/L NaCl, 2.5 mL of 20 mmol/L EDTA, 5 mL of 1 mol/L Tris-HCl (pH 8), and ddH_2_O to 50 mL. Approximately 0.1 g of each tissue sample was transferred to a centrifuge tube, and 500 µL of DNA lysis buffer was added. After grinding the samples with a tissue grinder, the samples were incubated at 55 °C for 5–8 h. Next, 200 µL of saturated NaCl was added, and the samples were vortexed for 10 s. The samples were then centrifuged at 12,000 × *g* for 10 min at 4 °C. The resulting supernatants were transferred to new centrifuge tubes, and equal volumes of isopropanol were added. After gently mixing up and down, the samples were allowed to stand at room temperature for 10 min and then centrifuged at 12,000 × *g* for 10 min at 4 °C. The supernatants were discarded, and the precipitates were gently washed once with 70% ethanol, and then centrifuged at 12,000 × *g* for 10 min at 4 °C. Finally, appropriate volumes of ddH_2_O were added to dissolve the precipitates. A NanoDrop 2000 (Agilent Technologies, Santa Clara, CA, USA) nucleic acid analyzer was used to assess DNA concentration and purity using OD_260/280_ (Additional file 2). To detect the ratio between mtDNA and nuclear DNA (nDNA), 50 ng DNA was analyzed by quantitative real-time PCR (qPCR) (Bio-Rad, California, USA).

### RNA extraction and qPCR

Total RNA was extracted from 19 samples (from all 5 stages D0, D7, D14, D21, and D28) using TRIzol (Invitrogen, Carlsbad, CA, USA), according to the manufacturer’s instructions [16]. Reverse transcription was performed with 2 µL of total RNA using Prime Script^TM^ RT reagent kit with gDNA Eraser (Tiangen, Beijing, China). qPCR was performed using the TB Green^®^ Premix Ex Taq^TM^ II (Takara) on a real-time PCR instrument (Bio-Rad, Richmond, CA, USA). The primers were designed using the Primer Premier 5.0 software. Primers used in this study were shown in Additional file 3. The 2^−ΔΔCT^ method was used to analyze the relative changes in gene expression normalized against goat β-Actin as an internal control.

### cDNA library construction and sequencing

cDNA libraries were constructed using the extracted total RNA samples. The cDNA libraries were then sequenced on an Illumina sequencing platform by Genedenovo Biotechnology Co., Ltd. (Guangzhou, China). The D14-4 sample failed quality inspection during cDNA library construction, and this sample was not included in the subsequent sequencing analysis. FASTP [23] software was used for quality control of the raw reads, and to filter low-quality data. HISAT2 [24] software was used to compare reads with the goat reference genome. R (http://www.r-project.org) was used to carry out principal component analysis (PCA) of the gene expression data, to evaluate the repeatability between samples, and to help exclude outlier samples. Differences in adipose tissue gene expression levels at D0, D7, D14, D21, and D28 were analyzed using DESeq2 software. The differentially expressed mRNAs with a false discovery rate (FDR) < 0.05 and |log_2_ (Fold Change)| > 1 were considered significant. STEM (Short Time-series Expression Miner) software was used to analyze and cluster the differentially expressed genes (DEGs) at the 5 time points. STEM was also used to visualize the expression patterns of genes at different stages after birth. The minimum variance for polygenic screening was 2, and the maximum number of trends was 20. Data were normalized using log_2_ (fragments per kilo-base of exon per million fragments mapped (FPKM)), and *P* < 0.05 was the screening range. GO analysis was used to classify the Biological Process, Cellular Component, and Molecular Function of DEGs. KOBAS software was used for the analysis of the KEGG (Kyoto Encyclopedia of Genes and Genomes) pathways, and the corrected *P*-value (FDR) cutoff was set at 0.05.

### Western blotting

The adipose tissues were triturated, and RIPA buffer (Beyotime, Shanghai, China) containing protease inhibitors was added. The samples were then incubated on ice for 30 min. Total protein concentrations were determined using a BCA protein analysis kit (Beyotime, Shanghai, China). Next, 25 µg of each protein was loaded, and the samples were separated by 12% SDS-PAGE (Bio-Rad). The resulting protein bands were then transferred to PVDF membranes for Western blotting. The PVDF membranes were blocked for 1 h at room temperature in 5% skimmed milk. Next, the membranes were incubated with primary antibodies (anti-UCP1, 1:1,000; anti-BAX, 1:1,000; anti-BCL2, 1:1,000; and anti-caspase-3, 1:1,000; Proteintech, Wuhan, China) at 4 °C overnight. The membranes were then washed with TBS with Triton (TBST) and incubated with the secondary antibody (1:1,000, Beyotime, Shanghai, China) at room temperature for 2 h. Finally, a bound antibody was detected using the Omni-ECL™ Femto Light Chemiluminescence kit (EpiZyme, Shanghai, China) on a chemiluminescence imager (Bio-Rad).

### Statistical analyses

All data were analyzed using SPSS 23.0 software and are expressed as mean ± standard error of the mean (SEM). The statistical significance was determined using *t*-tests and one-way ANOVA. Differences with a *P*-value < 0.05 were considered statistically significant.

## Results

### Mortality of Dazu black goat kids before weaning

Statistical analysis of the mortality data for Dazu black goat kids (before weaning) for 8 consecutive years (2011–2018) reveals that the mortality rate for winter-born kids was relatively high (Additional file 1: Fig. S2 and Additional file 4). This problem seriously impacts further development of Dazu black goat production, and a solution/reduction in the mortality rate of kids born in winter is urgently needed. How to solve this key production problem is essential to ensure the economic benefits of the Dazu black goat industry and the southern sheep industry.

### Thermographic evaluation of body surface temperatures

**Fig. 1:**
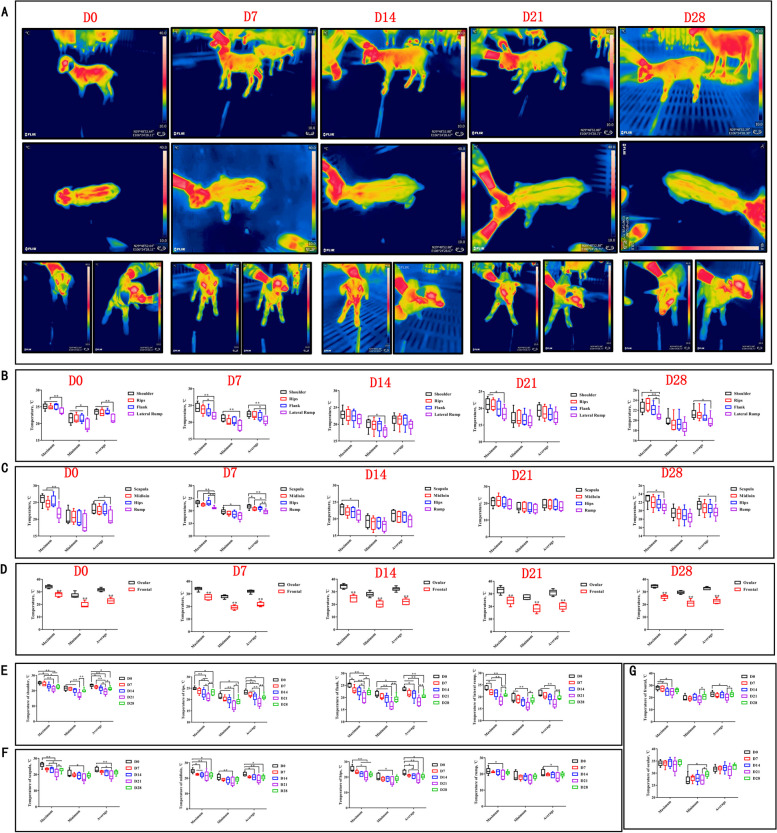
Thermographic images of D0–D28 goat. **A** Thermographic images were taken of goats from D0 to D28 after birth. Temperature statistics were collected for the left anatomical region (shoulder, ribs, flank, and lateral rump) (**B**), dorsal region (scapula, midloin, hips, and rump) (**C**), and head region (right ocular globe and forehead) (**D**). Temperature statistics were also collected for the left anatomical region (shoulder, ribs, flank, and lateral rump) (**E**), dorsal region (scapula, midloin, hips, and rump) (**F**), and head region (right ocular globe and forehead) (**G**) of goats from D0 to D28 after birth. ^*^P < 0.05, ^**^P < 0.01

To systematically analyze changes in thermogenic regions of goats during the postnatal period of D0–D28, we conducted thermographic evaluations of temperature changes in three anatomical regions: the left anatomical region (shoulder, ribs, flank, lateral rump), head region (right ocular globe and forehead), and dorsal region (scapula, midloin, hips, rump), details were showed in Additional file 1: Fig. S1. Results showed that the maximum temperatures of flank and hips were highest at D0, which were higher than lateral rump and rump respectively (*P* < 0.01) (Fig. 1A–C), shoulder and hips were highest at D7, which were higher than lateral rump and rump respectively (*P* < 0.01) (Fig. 1A–C), scapula was highest at D14, which were higher than rump (*P* < 0.05) (Fig. 1A–C), shoulder had the highest maximum temperature at D21, significantly higher than lateral rump (*P* < 0.05) (Fig. 1A–C), rips and scapula had the highest maximum temperature at D28, higher than lateral rump and rump respectively (*P* < 0.05) (Fig. 1A–C), and the right ocular globe temperature is significantly higher than the frontal temperature (Fig. 1A and D). A comparison of the postnatal stages D0–D28 showed that scapula, hips and flank were significantly higher at D0 than at D14 (*P* < 0.01), D21 (*P* < 0.01), and D28 (*P* < 0.01) (Fig. 1E and F), and that forehead temperatures at D0 also showed higher temperatures (Fig. 1G). Interestingly, there was an increase in temperature in the scapular and hips, which coincided with region of BAT deposition.

### Goat perirenal BAT whitening during the first month after birth

**Fig. 2:**
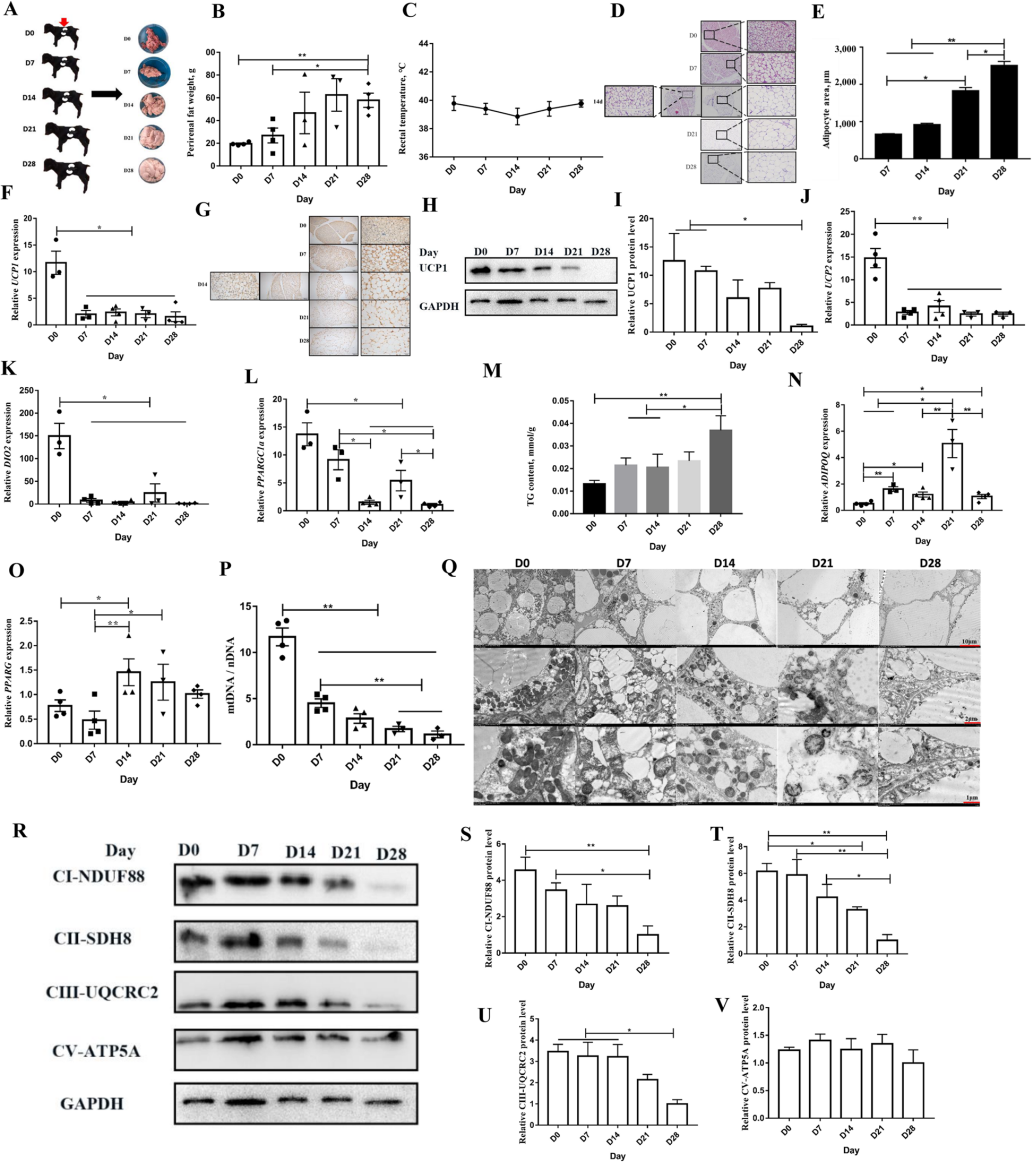
BAT changes to WAT during perirenal adipose tissue development. **A** Morphological comparison of perirenal adipose tissue. **B** Changes of perirenal adipose tissue weight. **C** Ratio of perirenal adipose tissue to bodyweight. **D** H&E staining of perirenal adipose tissue. **E** Statistics of perirenal adipose tissue area. **F–I** The mRNA expression (**F**), immunohistochemical staining (**G**), and protein expression (**H** and **I**) of UCP1. **J–L** Relative gene expression normalized to the geometric mean of *UCP2*, *DIO2*, and *PPARGC1a*. **M** The triglyceride content. **N **and** O** Relative gene expression normalized to the geometric mean of *ADIPOQ* (**N**) and *PPARG* (**O**). **P **and** Q** Perirenal adipose tissue mtDNA content (**P**) and observed by TEM (**Q**). **R–V** The expression levels of mitochondrial complex proteins (complexes I–V) were measured by Western blotting. ^*^P < 0.05, ^**^P < 0.01

The color of Dazu black goats perirenal adipose tissue was changed from reddish-brown in newborn D0 to milky white (white) in D28 and perirenal adipose tissue was significantly increased (Fig. 2A and B). However, there was no significant change in the adipose body ratio or rectal temperature over the same time period (Fig. 2C, Fig S3). According to the data of body height, body length, chest girth, chest width, and body weight, the Dazu black goat kids showed a rapid growth period from D0 to D28 (Additional file 1: Fig. S4A–E). H&E staining of D0–D28 perirenal adipose tissue revealed that the adipose tissue gradually changed from multilocular small lipid droplets at D0 to unilocular large lipid droplets at D28. The area of adipocytes also gradually increased during the D0–D28 period. At D7, the area of adipocytes was significantly lower than that at D21 (*P* < 0.05) and D28 (*P* < 0.01), and at D28, the area of adipocytes was significantly higher than that at D14 (*P* < 0.01) and D21 (*P* < 0.05) (Fig. 2D and E). The content of UCP1 gradually decreased with the change from BAT to WAT after birth (Fig. 2F–I). To explore the change in perirenal adipose tissue from BAT to WAT at the molecular level, we evaluated the mRNA expression levels of key thermogenesis-related genes (*UCP2*, *DIO2*, *PPARGC1a*, *PRDM16*, *CIDEA*, *C*/*EBPb*, and *C*/*EBPa*). *PRDM16* expression was significantly higher at D7 than at D0 (*P* < 0.01), D14 (*P* < 0.05), D21 (*P* < 0.05), and D28 (*P* < 0.05) (Additional file 1: Fig. S5A). *DIO2*, *UCP2*, *CIDEA*, *PPARGC1a*, *C*/*EBPb*, and *C*/*EBPa* were all highly expressed at D0 but downregulated at D28 (Fig. 2J–L and Additional file 1: Fig. S5A–D). Triglyceride content increased over the D0–D28 period, and the triglyceride content at D28 was significantly higher than that at D0 (*P* < 0.01), D7 (*P* < 0.05), and D14 (*P* < 0.05) (Fig. 2M). The mRNA expression levels of WAT marker genes (*PPARG*, *ADIPOQ*, *LPL*) were also measured by qPCR. *PPARG* expression at D0 was significantly lower than that at D14 (*P* < 0.05), and *PPARG* expression at D7 was significantly lower than that at D14 (*P* < 0.01) and D21 (*P* < 0.05) (Fig. 2O). *ADIPOQ* expression at D0 was significantly lower than that at D7 (*P* < 0.01), D14 (*P* < 0.05), D21 (*P* < 0.05), and D28 (*P* < 0.05). Interestingly, *ADIPOQ* expression was highest at D21, not D28 (Fig. 2N). *LPL* expression exhibited no statistical difference between D0 and D28 (*P* > 0.05) (Additional file 1: Fig. S5E). These results confirm that goat perirenal adipose tissue was mainly BAT at D0 and mainly WAT at D28. Another characteristic of BAT is that it contains more mitochondria (compared to WAT). While D0 perirenal adipose tissue had a high mitochondrial content, D28 perirenal adipose tissue had a relatively low mitochondrial content (Fig. 2P and Q). To confirm these findings, we evaluated the expression levels of mitochondrial complex proteins (complexes I–V). The expression of complex I protein at D28 was significantly lower than that at D0 (*P* < 0.01) and D7 (*P* < 0.05). Likewise, the expression of complex II protein at D28 was significantly lower than that at D0 (*P* < 0.01), D7 (*P* < 0.01), and D14 (*P* < 0.05). The expression of complex III protein at D28 was significantly lower than at D0 (*P* < 0.05), D7 (*P* < 0.05), and D148 (*P* < 0.05). Expression of complex IV was not detected, and there was no statistical difference in complex V (*P* > 0.05) (Fig. 2R–V). In conclusion, goat perirenal adipose tissue changes from BAT to WAT during the postnatal period (D0–D28). The BAT was predominant at D0, and the WAT was predominant at D28. This change is a spontaneous and normal biological process.

### Overview of RNA-sequencing and assessment of sequencing data quality

To analyze changes in gene expression as perirenal adipose tissue transitioned from BAT to WAT, we used RNA-seq to identify DEGs. After filtering out low-quality reads, we obtained 1.48 billion high-quality reads, with Q20 values above 95% and GC contents around 60% (Additional file 5 and Additional file 6). More than 82% of the reads matched the goat genome, and around 50% mapped to exon regions (Additional file 7 and Additional file 8), indicating the sequencing data were of high quality and suitable for bioinformatics analysis.

**Fig. 3:**
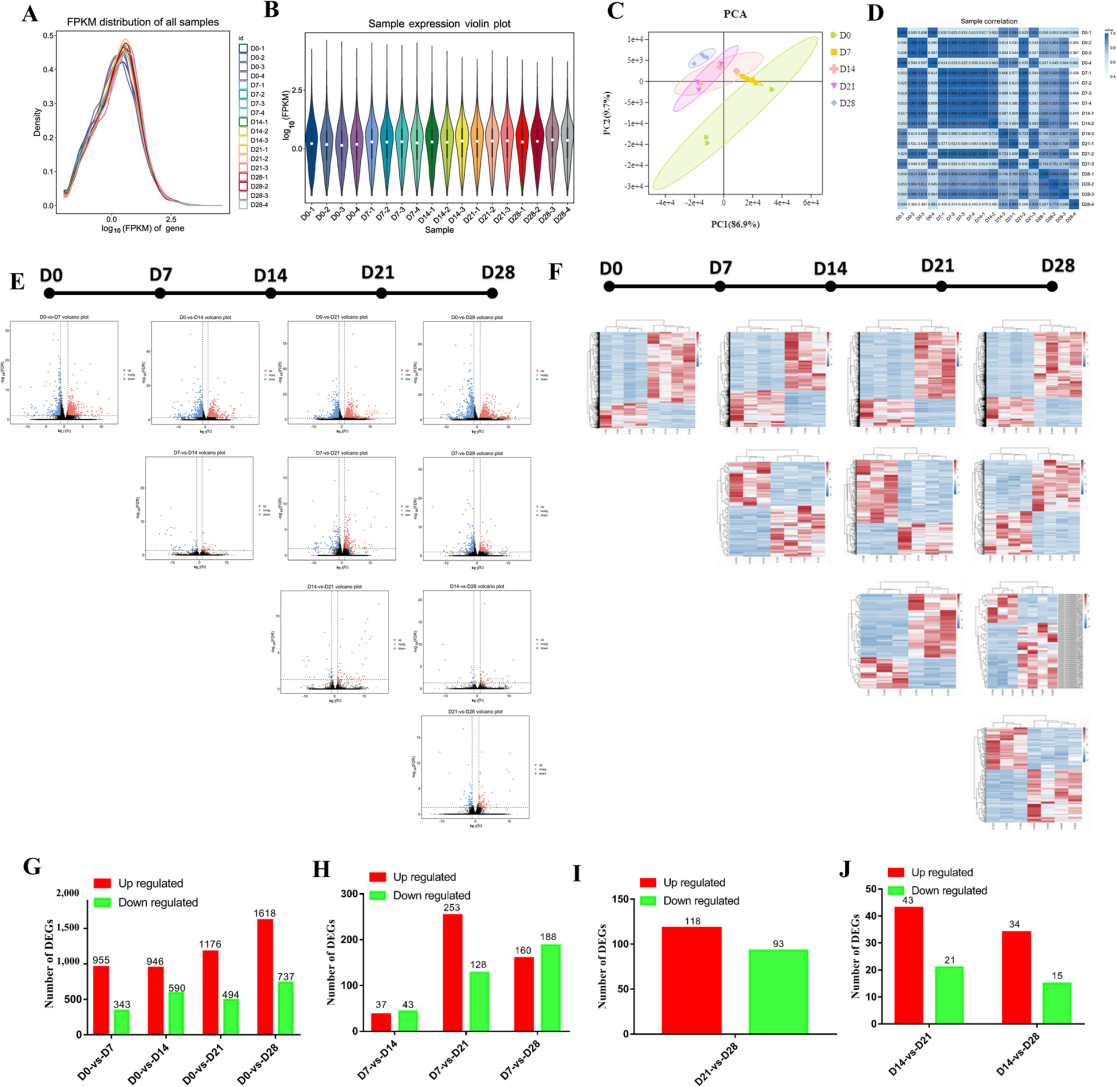
Overview of RNA-sequencing. **A** Gene expression abundance distribution map. **B** Violin plot of gene expression. **C** PCA analysis of 18 samples. **D** Sample correlation heat map. **E** Differential comparison volcano map. **F** Differential gene clustering heat map. **G** Differentially expressed mRNA statistics (up-regulated and down- regulated)

A total of 19,673 genes or transcripts were detected using RNA-seq, including 177 novel genes or transcripts. The FPKM values for all genes were obtained after correcting the sequencing depth and the length of the gene or transcript. A gene expression abundance distribution diagram is shown in Fig. 3A, and the gene expression violin chart is shown in Fig. 3B. To ensure the reliability and accuracy of sample selection, PCA was performed on the samples using the known mRNA expression results. The results demonstrate that perirenal adipose tissue undergoes a transitional change at D0, D7, D14, and D21, while D28 is clustered into a separate group (Fig. 3C). These results are consistent with the previous experimental results. In addition, the Pearson correlation coefficient of mRNA expression during the 5 stages was calculated using all 18 samples in the form of a heat map. While the differences within groups were small, the differences between groups were large. These results demonstrate that the repeatability of the sequencing was good, confirming that the sequencing samples can be used for differential expression analysis (Fig. 3D). Comparison analysis and statistical chart of the pairwise differences among the 5 time points (D0, D7, D14, D21, D28) are shown in Fig. 3E–J. For D0, there were 1,298 differentially expressed mRNAs from D0 vs. D7, 1,536 differentially expressed mRNAs from D0 vs. D14, 1,670 differentially expressed mRNAs from D0 vs. D21, and 2,355 differentially expressed mRNAs from D0 vs. D28 (Fig. 3G). For D7, there were 80 differentially expressed mRNAs from D7 vs. D14, 381 differentially expressed mRNAs from D7 vs. D21, and 348 differentially expressed mRNAs from D7 vs. D28 (Fig. 3H). For D14, there were 64 differentially expressed mRNAs from D14 vs. D21, and 49 differentially expressed mRNAs from D14 vs. D28 (Fig. 3I). For D21, there were 211 differentially expressed mRNAs from D21 vs. D28 (Fig. 3J). In summary, from D0 after birth in goat kids, the number of DEGs increased gradually during the change from BAT to WAT. Thus, D0 vs. D28 exhibits the largest number of DEGs. These results are in line with the previous phenotype data.

### Validation of RNA-seq by qPCR

**Fig. 4:**
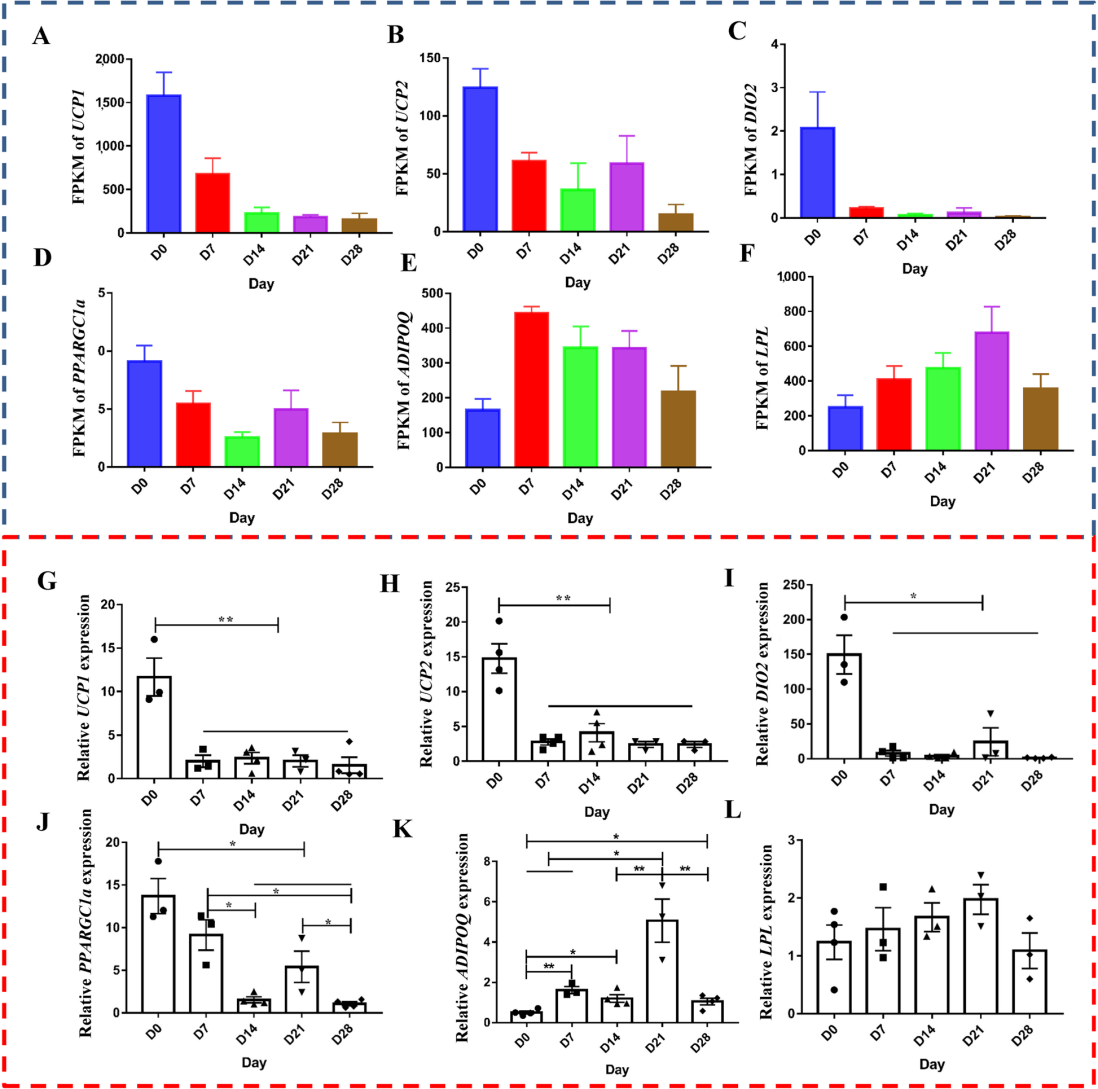
qPCR validation of RNA-seq data reliability. **A–F** mRNA expression levels measured by RNA-seq. **G–L** mRNA expression levels measured by qPCR. *P < 0.05, **P < 0.01

To verify the reliability of the RNA-seq data, 6 genes (*UCP1*, *UCP2*, *DIO2*, *PPARGC1a*, *ADIPOQ*, and *LPL*) were selected for validation. The results presented in Fig. 4A–L confirm that the RNA-seq data was consistent with the qPCR data. Thus, the RNA-seq data is reliable and may be used for subsequent bioinformatics analysis.

### Adipose tissue marker genes exhibit certain differences between species

**Fig. 5:**
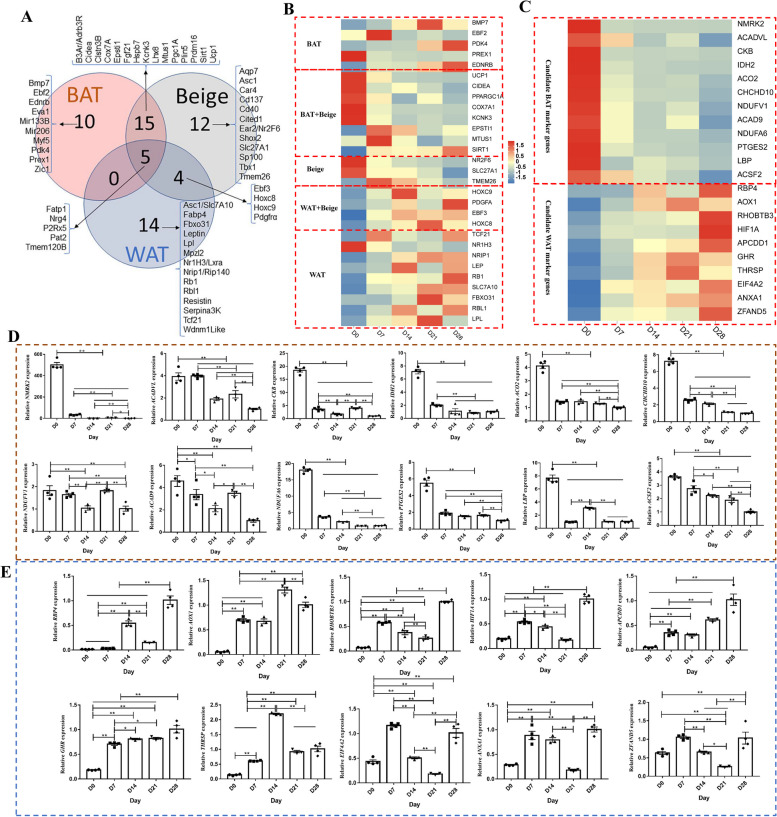
Candidate marker genes in adipose tissue. **A** Venn diagram of brown, beige, and white adipose marker genes. **B** Heat map of brown, beige, and white adipose marker genes from the RNA-seq data. **C** Heat map of BAT and WAT potential marker genes. **D** The expression levels of candidate BAT marker genes were measured by qPCR. **E** The expression levels of candidate WAT marker genes were measured by qPCR. *P < 0.05, **P < 0.01

With the advent of single-cell sequencing technology, marker genes can be used to mark cells and distinguish cell subsets. By identifying species-specific differences in the DEGs associated with the BAT to WAT change, we can better understand the different regulatory roles of key genes. Previous research on mice and human adipose tissues has identified some recognized BAT, beige and WAT marker genes [25] (Fig. 5A). The relevance of these marker genes to the change in goat is currently unknown. To address this, we used our RNA-seq data to assess the relevance of fat marker genes previously identified in human and mouse (and other species) for goat. According to our results, only one BAT marker gene, *PREX1*, was highly expressed at D0 in BAT. Although the expression levels of *BMP7*, *EBF2*, *PDK4*, and *EDNRB* were relatively high, changes in their expression levels did not conform with the phenotypic data. The expression levels of the remaining genes were relatively low or absent (Fig. 5B). The expression levels of *NR2F6* and *SLC27A1*, the only two accepted beige fat marker genes, conform with the phenotypic data. While *TMEM26* expression was relatively high, its expression does not conform with the phenotypic data. The expression levels of most of the remaining genes were relatively low or absent (Fig. 5B). The marker genes *UCP1*, *CIDEA*, *PPARGC1a*, *COX7A1*, and *KCNK3* were all co-expressed in brown and beige fat in line with the phenotype data. While the expression levels of *EPSTI1* and *SIRT1* were relatively high, their expression levels did not conform with the phenotypic data (Fig. 5B). Expression levels of the WAT marker genes *NRIP1*, *TCF21*, *LPL*, *LEP*, *HOXC8*, *HOXC9*, *EBF3*, *SLC7A10*, *FBXO31*, and *RBL1* were consistent with the phenotypic data (Fig. 5B). Taken together, these putative markers for BAT, beige, and WAT do not seem to fully fit the definitions of BAT and WAT in goats.

To further study goat fat development and its regulation, we also screened potential marker genes suitable for goat BAT and WAT using the screening criteria of FPKM > 100 and fold change > 5. We identified 12 potential marker genes (*NMRK2*, *ACADVL*, *CKB*, *IDH2*, *ACO2*, *CHCHD10*, *NDUFV1*, *ACAD9*, *NDUFA6*, *PTGES2*, *LBP*, and *ACSF2*) for goat BAT and 10 potential marker genes (*RBP4*, *AOX1*, *RHOBTB3*, *HIF1A*, *APCDD1*, *GHR*, *THRSP*, *EIF4A2*, *ANXA1*, and *ZFAND5*) for goat WAT (Fig. 5C–E). However, since these screened marker genes have yet to be mechanistically validated, they should be regarded as reference only. Further investigation is needed to confirm that these genes do indeed serve as marker genes for BAT and WAT in goats.

### Mining of new candidate genes for thermogenesis in BAT

**Fig. 6:**
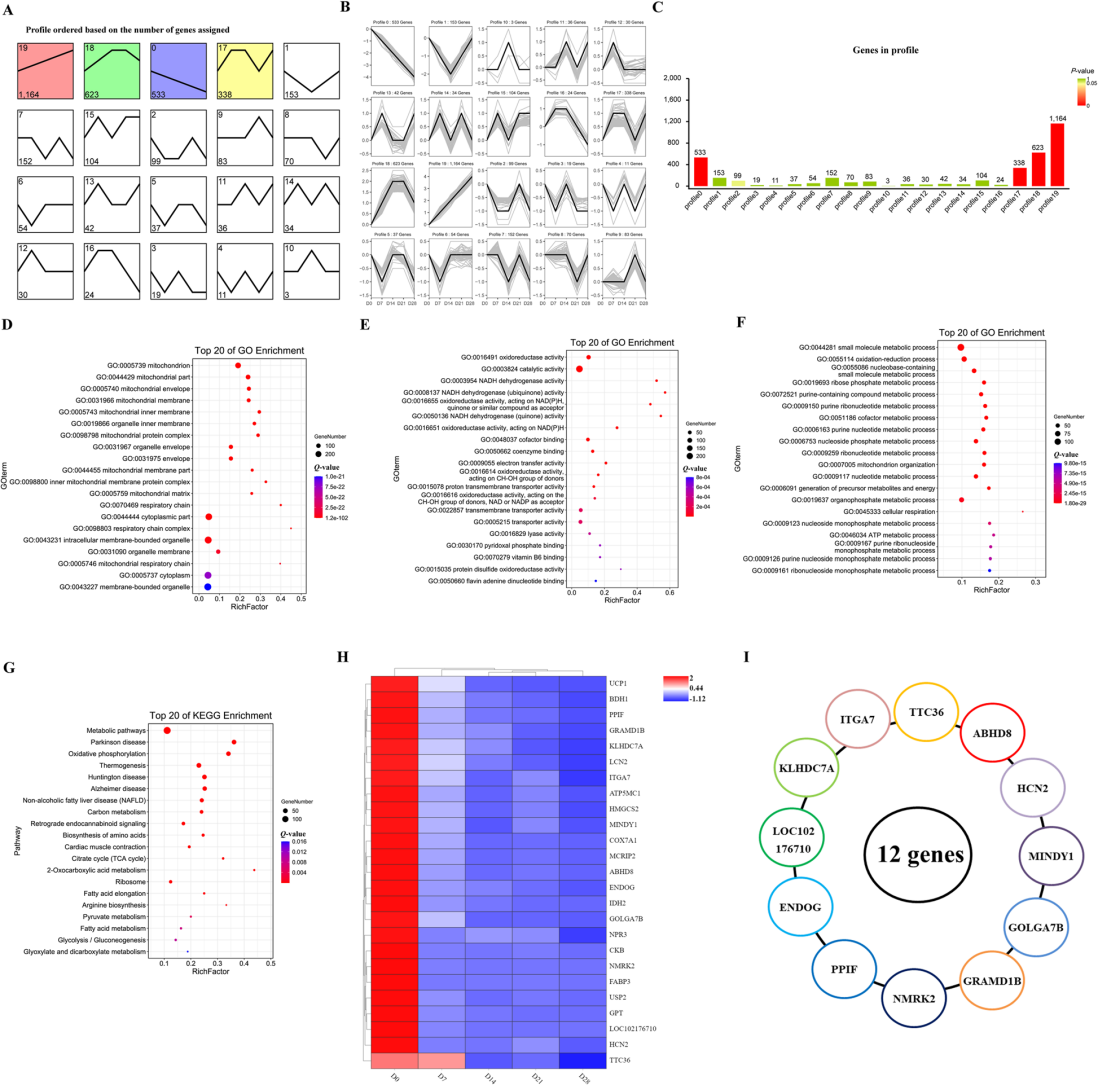
Selection of new candidate genes for thermogenesis in BAT. **A** Distribution trend of differentially expressed genes, color indicates a significant difference (*P* < 0.05), gray indicates no significant difference (*P* > 0.05). **B** Time series line of differentially expressed genes. **C** Statistics of the number of differentially expressed mRNAs. **D** GO bubble map of cellular components. **E** GO bubble map of molecular functions. **F** GO bubble map of biological processes. **G** Bubble map of KEGG pathway analysis. **H** Heatmap of Top 25 differentially expressed mRNAs. **I** The differentially expressed mRNA patterns of 12 novel genes

Analysis of DEGs in perirenal adipose tissue from D0 to D28 revealed 20 different expression trends, with four showing significant differences (Fig. 6A). Interestingly, these four expression trends were enriched with most of the DEGs (Fig. 6C) and could be roughly categorized as up-regulated or down-regulated (Fig. 6B). To identify new genes related to BAT thermogenesis regulation, we analyzed genes that were linearly down-regulated (profile 0) during the D0–D28 time period and performed functional enrichment analysis, revealing involvement in metabolic and tricarboxylic acid cycle signaling pathways (Fig. 6D–G). From the subset of genes with high FPKM values and significant fold changes, we identified 12 novel candidate genes (*NMRK2*, *PPIF*, *ENDOG*, *LOC102176710*, *KLHDC7A*, *ITGA7*, *TTC36*, *ABHD8*, *HCN2*, *MINDY1*, *GOLGA7B*, and *GRAMD1B*) related to BAT thermogenesis or adipocyte differentiation (Fig. 6H and I). However, further studies are needed to investigate the functions of these genes in goat BAT thermogenesis.

### Potential mechanisms of BAT to WAT changes

**Fig. 7:**
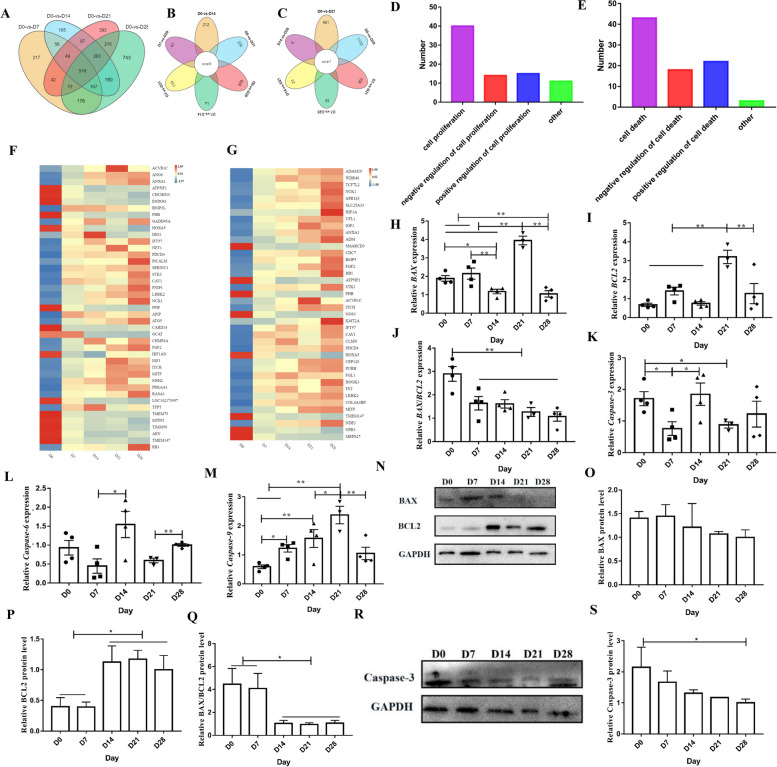
Potential mechanisms of BAT to WAT changes. **A–C** Venn diagram of different groups. **D** Number of genes regulating cell death. **E** Number of genes regulating cell proliferation. **F** Heat map of gene expression regulating cell death from RNA-seq data. **G** Heat map of gene expression regulating cell proliferation from the RNA-seq data. **H–M** Relative gene expression normalized to the geometric mean of *BAX*, *BCL2*, *BAX*/*BCL2*, caspase-3, caspase-6, and caspase-9. **N–S** BAX, BCL2, and caspase-3 protein expression levels were measured by Western blotting. ^*^P < 0.05, ^**^P < 0.01

In total, 519 differentially co-expressed genes were identified in the first group (Fig. 7A, Additional file 9), 9 differentially co-expressed genes in the second group (Fig. 7B, Additional file 10), and 1 differentially co-expressed gene in the third group (Fig. 7C, Additional file 11). GO enrichment analysis was then performed on the common DEGs (Additional file 12 and Additional file 13). No genes involved in the regulation of cell death and proliferation were identified in Fig. 7B and C. Analysis of the genes in Fig. 7A reveals that 43 genes identified are involved in the regulation of cell death (Fig. 7D and F). Of these, 17 genes (*ACVR1C*, *ANO6*, *ANXA1*, ATP5IF1, *CHCHD10*, *ENDOG*, *BNIP3L*, *PHB*, *GADD45A*, *HOXA5*, *IDO1*, *IFT57*, *NET1*, *PDCD4*, *PICALM*, *SERINC3*, and *STK3*) were enriched in positively regulated cell death pathways, 13 genes (*APIP*, *ATG5*, *CARD14*, *GCAT*, *CHMP4A*, *FGF2*, *HIF1AN*, *IGF1*, *ITCH*, *MITF*, *MSH2*, *PRKAA*, and *RASA1*) were enriched in negatively regulated cell death pathways, and 5 genes (*CAV1*, *FNIP1*, *LRRK2*, *NCK1*, and *PPIF*) were enriched in both positive and negatively regulated cell death pathways, The remaining genes were enriched in other pathways that regulate cell death. In addition, 40 genes were involved in the regulation of cell proliferation (Fig. 7E and G). Of these, 13 genes (*ADAM10*, *WDR48*, *TCF7L2*, *NCK1*, *GPR183*, *SLC25A33*, *HIF1A*, *UFL1*, *IGF1*, *ANXA1*, *ADM*, *SMARCD3*, and *CDC7*) were enriched in positively regulated cell proliferation pathways, 12 genes (*RB1*, *ATP5IF1*, *STK3*, *PHB*, *ACVR1C*, *ITCH*, *NOS3*, *KMT2A*, *IFT57*, *CAV1*, *CLMN*, and *PDCD4*) were enriched in negatively regulated cell proliferation pathways, and 2 genes (*BMP5*, *FGF2*) were enriched in both positive and negative regulatory cell proliferation pathways. It should be noted that the genes regulating cell death and proliferation did not show a continuous high expression during D0, D7, D14, D21, and D28 (Fig. 7D and E). The observed change from BAT to WAT during the development of perirenal adipose tissue over the D0–D28 period can be considered a spontaneous and normal biological process. In general, normal physiological processes do not induce high rates of tissue death and proliferation.

In addition, we assessed changes in the expression of known apoptosis-related genes (*BAX*, *BCL2*, caspase-3, caspase-6, caspase-9) in the D0–D28 period. *BAX* mRNA expression was significantly lower at D28 compared with D0 (*P* < 0.01), D7 (*P* < 0.01), and D21 (*P* < 0.01) (Fig. 7H). *BCL2* expression was highest at D21, where it was significantly higher than D0 (*P* < 0.01), D7 (*P* < 0.01), D14 (*P* < 0.01), and D28 (*P* < 0.01) (Fig. 7I). The *BAX*/*BCL2* ratio at D0 was significantly higher than that at D7 (*P* < 0.05), D14 (*P* < 0.05), D21 (*P* < 0.05), and D28 (*P* < 0.05) (Fig. 7J). While there was no significant difference in BAX protein expression between the groups (*P* > 0.05), BCL2 protein expression was significantly higher at D14, D21, and D28 than at D0 (*P* < 0.05) and D7 (*P* < 0.05). The BAX/BCL2 ratio was also significantly higher at D0 and D7 compared with D14 (*P* < 0.05), D21 (*P* < 0.01), and D28 (*P* < 0.01) (Fig. 7G–Q). The expression levels of caspase-3 and caspase-6 did not exhibit regular change (Fig. 4K and L), while the expression levels of caspase-9 gradually increased at D0, D7, D14, and D21. Caspase-9 expression at D0 was significantly lower than that at D7 (*P* < 0.05), D14 (*P* < 0.01), and D21 (*P* < 0.01), caspase-9 expression at D7 was significantly lower than that at D21 (*P* < 0.01), and caspase-9 expression at D14 was significantly lower than that at D21 (*P* < 0.05) (Fig. 7M). It was also noted that caspase-3 protein expression levels were not completely consistent with caspase-3 mRNA expression levels, and the difference was significantly higher at D0 compared with D28 (*P* < 0.05) (Fig. 7R and S).

Taken together, the observed expression changes during the change of BAT to WAT during D0–D28 perirenal adipose tissue development do not involve large-scale death of brown adipocytes and subsequent white adipocyte proliferation. Our results suggest that the changes in expression related to apoptosis may play a limited regulatory role in this change process.

## Discussion

In southern China, winter is characterized by low temperatures and high humidity, which can lead to hypothermia, one of the most common causes of mortality in neonatal southern goats [26, 27]. Despite the milder winter in the south compared to the north, low temperatures are often overlooked, resulting in a high mortality rate in pre-weaning kids, as observed in Dazu black goats, as reported previously [5]. With global warming and associated extreme weather changes, it is important to pay more attention to the economic losses caused by cold stress in the goat industry during winter. Thermal imaging is a non-contact, non-destructive, and highly sensitive temperature detection method that offers a potential non-invasive approach for studying thermogenesis in newborn animals [28]. Infrared thermography can detect thermal loss and BAT-associated thermogenesis more effectively than core body temperature measurements and has been effectively utilized for thermal mapping of sheep [20, 29, 30]. However, there are no systematic studies on the thermoregulatory responses and thermogenic regions of newborn goats in the literature. Therefore, investigating the thermoregulatory capacity of goat kids at different stages after birth is critical for broadening the understanding of thermal biology during an extremely sensitive stage of life. In our present study, histological analysis reveals that the perirenal adipose tissue of Dazu black goats changed from BAT to WAT in the period from D0 to D28. Interestingly, there was an increase in temperature in the hips at D0, which coincided with region of perirenal BAT deposition. These results are consistent with previous reports [20, 28]. For ethical reasons, the goats were not sheared in this experiment. However, post-shearing thermography to assess changes in the regions of thermogenesis on the goats’ body surface may be closer to the real body temperature.

The adipose organ is regulated by the environment, temperature, dietary habits, and hormones [31]. Adipose plasticity can occur through the proliferation and differentiation of stem cells and through the direct transformation of mature adipocytes [32, 33]. Indeed, mature adipocytes are known to change their phenotype and function by reprogramming following the appropriate stimuli (e.g., cold exposure or β3-adrenoreceptor agonists) [31–33]. Because BAT uses triglycerides to generate thermogenesis, resist cold [9], and combat obesity [34], an increase in BAT could improve survival in young animals. While perirenal adipose tissue in newborn sheep and goats is composed of brown tissue at birth, and the expression of *UCP1* peaks at birth, the BAT gradually changes to WAT [8, 10, 11, 19]. The same phenomenon occurs in rabbit interscapular adipose tissue [14]. In our present study, histological analysis reveals that the perirenal adipose tissue of Dazu black goats changed from BAT to WAT in the period from D0 to D28. While BAT was predominant at D0, WAT was predominant at D28. Several thermogenesis-related genes [35], including *UCP1*, *CIDEA*, and *PPARGC1a*, which are all highly expressed in BAT, were highly expressed at D0 in perirenal adipose tissue. The mitochondrial content was also observed to decrease with the change from BAT to WAT. These results are consistent with previous reports [10], and they suggest that this BAT to WAT change is a spontaneous and normal biological process. However, this change is not consistent with the changes reported in mice [36].

Numerous studies of adipose tissue have also identified putative marker genes for BAT, beige, and WAT [25, 37–42]. While mouse studies have often been employed to understand this change in other species, the change from BAT to WAT in the perirenal adipose tissue organ of the goat was not consistent with that in mice [43]. Therefore, it is important to explore species-specific differences in reported adipocyte marker genes, especially if the aim is to conduct research on the regulation of adipocyte development in another species (e.g., goat). Brown adipocyte marker genes *EDNRB* [44], *SIRT1* [25], *EPSTI1* [25, 40], *BMP7* [45], and *PDK4* [25] were all highly expressed in WAT from goat kids but not in BAT. This reverse pattern of marker gene expression in adipose tissue was previously observed when comparing humans and mice [43]. In addition, *ZIC1* [42] and *MYF5* [25], two brown adipocyte-specific genes, were not expressed in goat BAT. These results are consistent with a previous study in sheep [10]. The expression of *MPZL2* [46], a marker gene of WAT, was undetectable during the D0–D28 period. The expression levels of *LHX8* [35] and *FGF21* [47], two marker genes of BAT, did not change over the D0–D28 period. *UCP1*, *CIDEA*, *PREX1*, *COX7A1*, *KCNK3*, and *PPARGC1a* (and several other marker genes) were all highly expressed in D0 perirenal adipose tissue (i.e., in brown tissue). *NRIP1*, *TCF21*, *LPL*, LEP, *HOXC8*, *HOXC9*, *EBF3*, *SLC7A10*, *FBXO31*, and *RBL1* (white tissue marker genes) were all highly expressed in D28 perirenal adipose tissue [25, 37–42]. The above results provide confirmation of a change in goat perirenal adipose tissue from BAT to WAT over the D0–D28 period. Moreover, the results highlight differences in adipose tissue marker genes between species, which should be studied more closely. Thus, key differentially expressed adipose tissue genes between species should be the focus of future adipose tissue research.

To date, there is no systematic description of adipose tissue marker genes in goats. Therefore, we functionally screened 12 potential marker genes (*NMRK2*, *ACADVL*, *CKB*, *IDH2*, *ACO2*, *CHCHD10*, *NDUFV1*, *ACAD9*, *NDUFA6*, *PTGES2*, *LBP*, and *ACSF2*) for goat BAT identified from our RNA-seq data. *NMRK2* has been widely reported to be involved in biological processes such as body metabolism and aging [48, 49]. *ACADVL* is a β-oxidation-related gene [50]. Selective inactivation of *CKB* reduces the thermogenic capacity of fat, increases obesity propensity, and disrupts glucose homeostasis [51]. Knockout of *IDH2* affects mitochondrial function in BAT, causing BAT whitening, which in turn contributes to obesity in high-fat-fed mice [52]. *ACO2* is highly expressed in human and mouse BAT [43]. *CHCHD10*, *NDUFV1*, *ACAD9*, and *NDUFA6* are all mitochondria-related genes (here, we note that the mitochondrial number was significantly changed in our TEM study). *ACSF2* is involved in metabolic pathways such as the tricarboxylic acid cycle [53]. While *LBP* is a negative regulator of adipose tissue browning in mouse and human [54], our results indicate that it is highly expressed in goat BAT (which is the reverse of the expression pattern observed in human and mouse). Thus, *LBP* may be an attractive candidate for future studies in goat adipose tissue at a later stage. Similarly, the role of *PTGES2* in goat BAT should be further investigated. Likewise, 10 potential marker genes (*RBP4*, *AOX1*, *RHOBTB3*, *HIF1A*, *APCDD1*, *GHR*, *THRSP*, *EIF4A2*, *ANXA1*, *ZFAND5*) for goat WAT were identified. *RBP4*, *GHR*, *ANXA1*, and *HIF1A* are all strongly associated with obesity [55–58]. *APCDD1* is a novel regulator that promotes adipocyte differentiation [59]. *THRSP* expression is closely correlated with bovine intramuscular fat content [60]. While we have screened out several potential marker genes for goat adipose tissue, their functions in goat adipose tissue need to be further explored. In addition, we also found that 25 genes may be involved in the regulation of BAT thermogenesis, including *UCP1* [25], *COX7A1* [25, 61], *FABP3* [61], *GPT* [62], *CKB* [51], *IDH2* [53], *LCN* [63], *HMGCS2* [64], *MCRIP2* [65], and *NPR3* [66]. All 25 genes have previously been reported to be involved in the regulation of fat metabolism. This further demonstrates the reliability of our screening data. Whether *NMRK2*, *PPIF*, *ENDOG*, *LOC102176710*, *KLHDC7A*, *ITGA7*, *TTC36*, *ABHD8*, *HCN2*, *MINDY1*, *GOLGA7B*, and *GRAMD1B* have regulatory roles in goat BAT thermogenesis will be the focus of our later research.

The change from BAT to WAT has been reported in goats [11, 19], sheep [8, 10], and rabbits [14], and some scholars have proposed that this process involves transformation [10, 11]. However, a more detailed and systematic account is needed. According to the widely accepted paradigm, brown adipocytes and muscle cells originate from a common Myf5^+^ precursor cell, whereas white adipocytes and beige adipocytes derive from a Myf5^−^ precursor cell [67]. Therefore, the conversion of BAT to WAT, as proposed, appears to contradict this paradigm. Notably, this conversion is not observed in mice, whereas in humans, the BAT present in childhood is largely lost in adulthood [17], a pattern reminiscent of that seen in goats, sheep, and rabbits. To shed light on the underlying mechanisms of this phenomenon, we have advanced three hypotheses: (1) Brown adipocytes die and white adipocytes proliferate; (2) BAT transdifferentiates into WAT; and (3) low- and high-thermogenic brown adipocyte subpopulations coexist [68], with the high-thermogenic subpopulations prevailing at D0, followed by a shift to subpopulations associated with high-fat deposition. These hypotheses require further testing through a variety of experiments to uncover the key regulatory factors and mechanisms. Our results suggest that the change from BAT to WAT that occurs during perirenal adipose tissue development in D0–D28 goat kids is not due to brown adipocyte death and white adipocyte proliferation. However, apoptosis may play a limited regulatory role in this change process. This result is consistent with previous research on sheep [10]. In conclusion, our results indicate that the change process from BAT to WAT in the perirenal adipose tissue of goat kids does not involve the programmed death of brown adipocytes. We believe that brown adipocytes may have undergone transdifferentiation, as terminal cell conversions are frequently reported [69]. Alternatively, it is possible that a coexistence of high thermogenic and high fat depositing adipocyte subpopulations is present, given the known heterogeneity of cells in adipose tissue [68]. However, we acknowledge that more research is required on the mechanism by which this phenomenon occurs.

## Conclusions

**Fig. 8:**
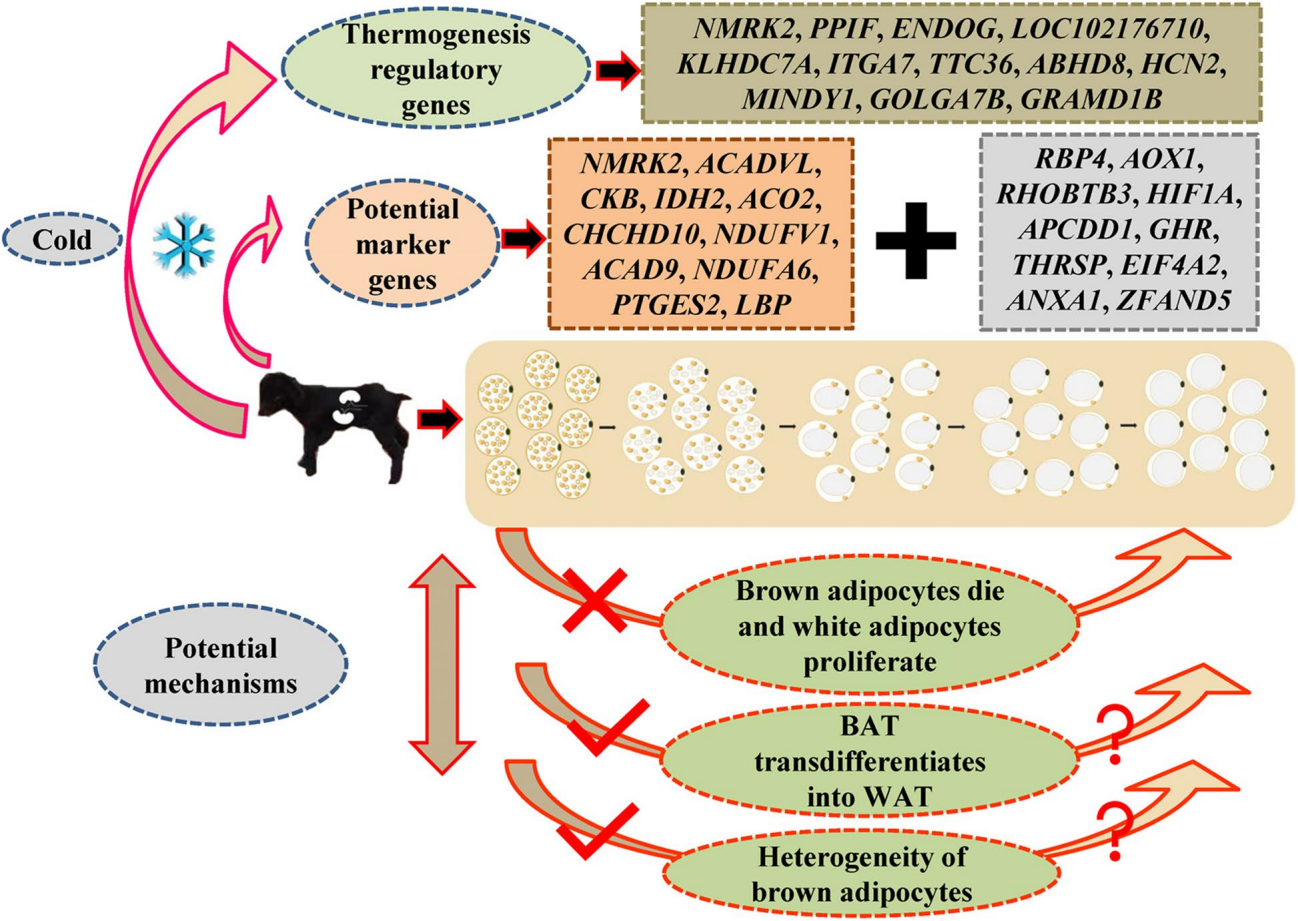
Summary of this study. The D0–D28 goat perirenal adipose tissue has a change process from BAT to WAT. This change process is not caused by the programmed death of brown adipocytes and the proliferation of white adipocytes. In addition, known adipose tissue marker genes demonstrate interspecies differences. We also identified 12 potential marker genes (*NMRK2*, *ACADVL*, *CKB*, *IDH2*, *ACO2*, *CHCHD10*, *NDUFV1*, *ACAD9*, *NDUFA6*, *PTGES2*, *LBP*, and *ACSF2*) for goat BAT and 10 potential marker genes (*RBP4*, *AOX1*, *RHOBTB3*, *HIF1A*, *APCDD1*, *GHR*, *THRSP*, *EIF4A2*, *ANXA1*, and *ZFAND5*) for goat WAT. Twelve new candidate genes (*NMRK2*, *PPIF*, *ENDOG*, *LOC102176710*, *KLHDC7A*, *ITGA7*, *TTC36*, *ABHD8*, *HCN2*, *MINDY1*, *GOLGA7B*, and *GRAMD1B*) regulating brown fat thermogenesis were identified

In conclusion, our study reveals that perirenal BAT is essential for regulating thermoregulation in newborn goat kids. Interestingly, we identified notable differences in the expression of adipose tissue marker genes between species, and we highlighted 12 potential marker genes for goat BAT and 10 for goat WAT. Our findings also led to the identification of 12 new candidate genes that regulate BAT thermogenesis (Fig. 8). Importantly, the change from BAT to WAT appears not to involve a large-scale death of brown adipocytes and subsequent proliferation of white adipocytes. However, further research is needed to clarify whether this change in BAT to WAT is due to the presence of transdifferentiation in mature adipocytes or the heterogeneity of adipocytes, which leads to low- and high-thermogenic brown adipocyte subpopulations coexisting.

### Supplementary Information

**Additional file 1**: **Fig. S1.** Illustrative thermographic images of the evaluation of the left anatomical region (shoulder, rips, flank, lateral rump) (**A**), dorsal region (scapula, midloin, hips, rump) (**B**), forehead region (**C**), right ocular globe (**D**).** Fig. S2. **Pre-weaning kid mortality statistics (2011–2018). **Fig. S3.** Ratio of perirenal adipose tissue to bodyweight. **Fig. S4.** Growth performance and rectal temperature testing. **A** Body height; **B** Body length; **C** Chest girth; **D** Chest width; **E** Bodyweight; **F** Rectal temperature. **Fig. S5.** Relative gene expression normalized to geometric mean of *PRDM16*, *CIDEA*,*C*/*EBPb*, *C*/*EBPa*, and *LPL*. ^*^P < 0.05, ^**^P < 0.01

**Additionalfile 2: Table S1. **The concentration and purity of total DNA in goat adipose tissue.

**Additional file 3: Table S2. **Primers for qPCR of adipose tissue in goats.

**Additional file 4: Table S3.** Pre-weaning kid mortality statistics (2011–2018).

**Additional file 5: Table S4. **Overview of Sequencing Data.

**Additional file 6: Table S5. **Bases mass analysis.

**Additional file 7: Table S6.** Reference genome alignment.

**Additional file 8: Table S7.** Comparison of reference area.

**Additional file 9: Table S8. **D0 vs D7 and D0 vs D14 and D0 vs D21 and D0 vs D28 co-expressed genes.

**Additional file 10: Table S9.** D0 vs D14, D21, D28, and D7 vs D14, D21, D28 co-expressed genes.

**Additional file 11: Table S10.** D0 vs D21, D28 and D7 vs D21, D28 and D14 vs D21, D28 co-expressed genes.

**Additional file 12: Table S11.** GO enrichment analysis of 519 genes.

**Additional file 13: Table S12.** GO enrichment analysis of 9 genes.

## Data Availability

The data analyzed during the current study are available from the corresponding author on reasonable request.

## Abbreviations

ACAD9: Acyl-CoA dehydrogenase family member 9
ACADVL: Acyl-CoA dehydrogenase very long chain
ACO2: Aconitase 2
ACSF2: Acyl-CoA synthetase family member 2
ACTIN: Actin beta
ADIPOQ: Adiponectin:C1Q and collagen domain containing
ANXA1: Annexin A1
AOX1: Aldehyde oxidase 2
APCDD1: APC down-regulated 1
BAT: Brown adipose tissue
BAX: BCL2 associated X:apoptosis regulator
BCL2: BCL2 apoptosis regulator
C/EBPA: CCAAT enhancer binding protein alpha
C/EBPB: CCAAT enhancer binding protein beta
CHCHD10: Coiled-coil-helix-coiled-coil-helix domain containing 10
CIDEA: Cell death inducing DFFA like effector a
CKB: Creatine kinase B
DEGs: Differentially expressed genes
DIO2: Iodothyronine deiodinase 2
EIF4A2: Eukaryotic translation initiation factor 4A2
FDR: False discovery rate
FPKM: Fragments per kilo-base of exon per million fragments mapped
GHR: Growth hormone receptor
H&E: Hematoxylin-eosin
HIF1A: Hypoxia inducible factor 1 subunit alpha
IDH2: Isocitrate dehydrogenase (NADP(+)) 2
KEGG: Kyoto Encyclopedia of Genes and Genomes
LBP: Lipopolysaccharide binding protein
LPL: Lipoprotein lipase
mtDNA: Mitochondrial DNA
nDNA: Nuclear DNA
NDUFA6: NADH:ubiquinone oxidoreductase subunit A6
NDUFV1: NADH:ubiquinone oxidoreductase core subunit V1
NMRK2: Nicotinamide riboside kinase 2
PCA: Principal component analysis
PPARG: Peroxisome proliferator activated receptor gamma
PPARGC1a: PPARG coactivator 1 alpha
PRDM16: PR/SET domain 16
PTGES2: Prostaglandin E synthase 2
qPCR: Quantitative real-time PCR
RBP4: Retinol binding protein 4
RHOBTB3: Rho related BTB domain containing 3
STEM: Short Time-series expression Miner
TBST: TBS with Triton
TEM: Transmission electron microscope
TG: Triglyceride
THRSP: Thyroid hormone responsive
UCP1: Uncoupling protein 1
UCP2: Uncoupling protein 2
WAT: White adipose tissue
ZFAND5: Zinc finger AN1-type containing 5
