# Supplementary material for: Global gene expression profiling of perirenal brown adipose tissue whitening in goat kids reveals novel genes linked to adipose remodeling
Source: J Anim Sci Biotechnol. 2024 Mar 14;15:47. doi: 10.1186/s40104-024-00994-w (PMC10938744; doi:10.1186/s40104-024-00994-w)
Supplement: Supplementary file 1 — Additional file 1: Fig. S1. Illustrative thermographic images of the evaluation of the left anatomical region (shoulder, rips, flank, lateral rump) (A), dorsal region (scapula, midloin, hips, rump) (B), forehead region (C), right ocular globe (D). Fig. S2. Pre-weaning kid mortality statistics (2011–2018). Fig. S3. Ratio of perirenal adipose tissue to bodyweight. Fig. S4. Growth performance and rectal temperature testing. A Body height; B Body length; C Chest girth; D Chest width; E Bodyweight; F Rectal temperature. Fig. S5. Relative gene expression normalized to geometric mean of PRDM16, CIDEA,C/EBPb, C/EBPa, and LPL. *P < 0.05, **P < 0.01 [file 40104_2024_994_MOESM1_ESM.docx]

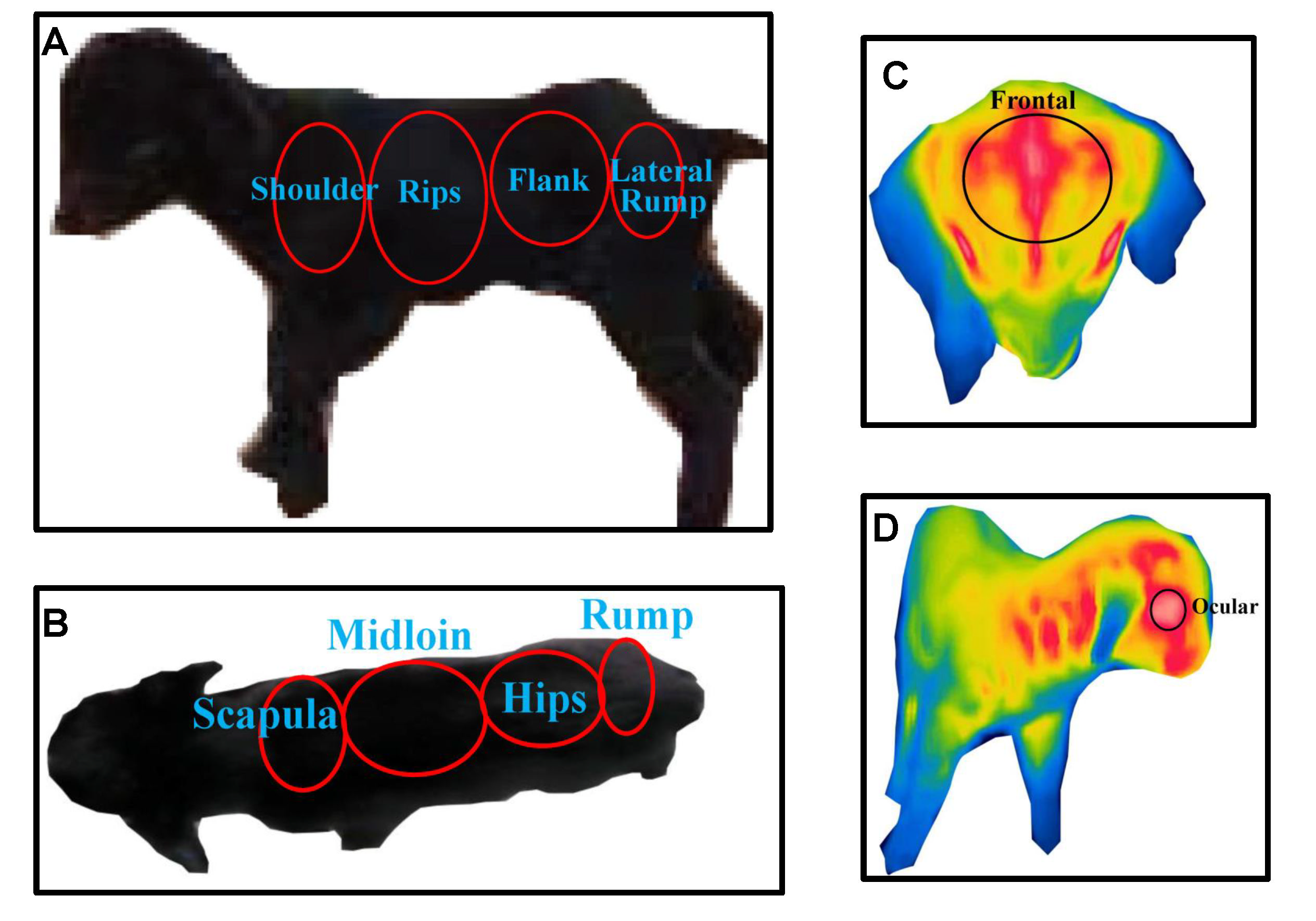


**Fig. S1** Illustrative thermographic images of the evaluation of the left anatomical region (shoulder, rips, flank, lateral rump) (**A**), dorsal region (scapula, midloin, hips, rump) (**B**), forehead region (**C**), right ocular globe (**D**)


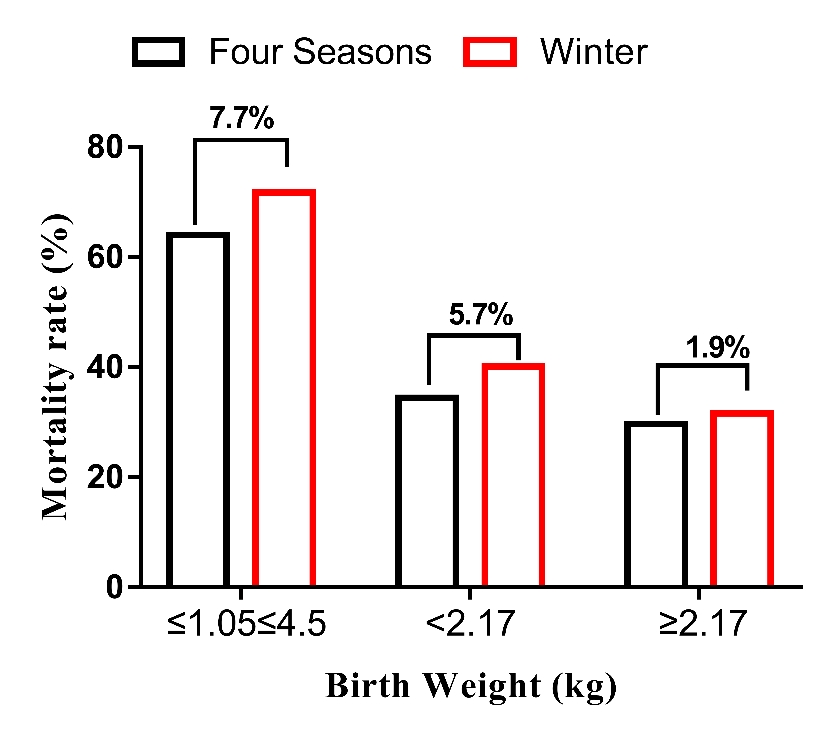


**Fig. S2** Pre-weaning kid mortality statistics (2011–2018)


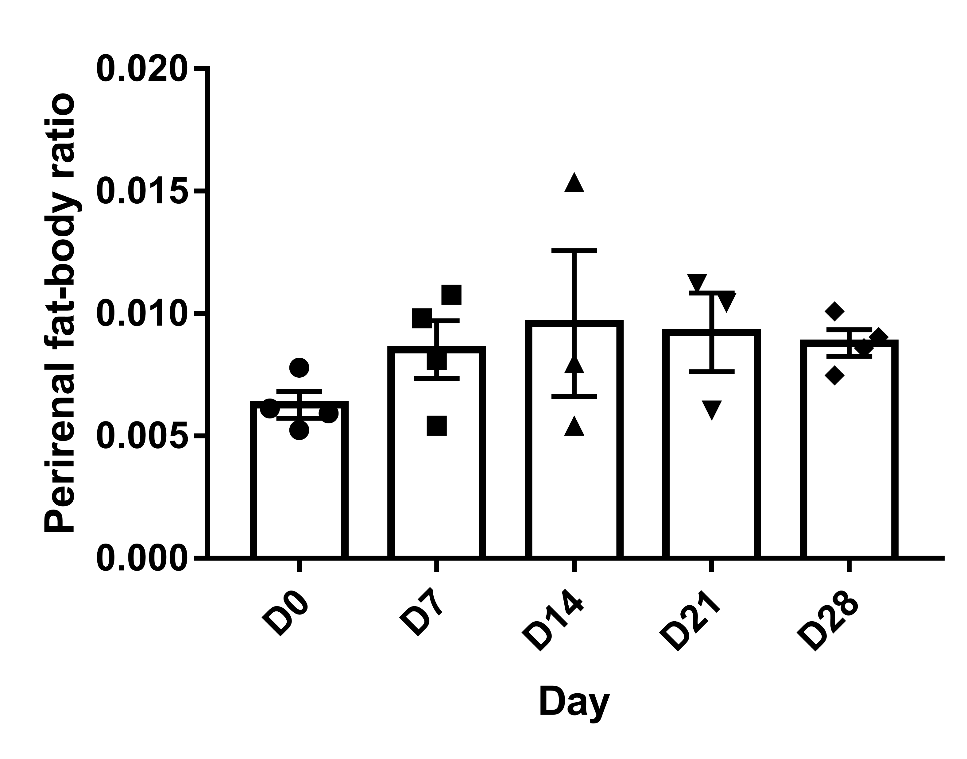


**Fig. S3** Ratio of perirenal adipose tissue to bodyweight. ^*^*P <* 0.05, ^**^*P <* 0.01


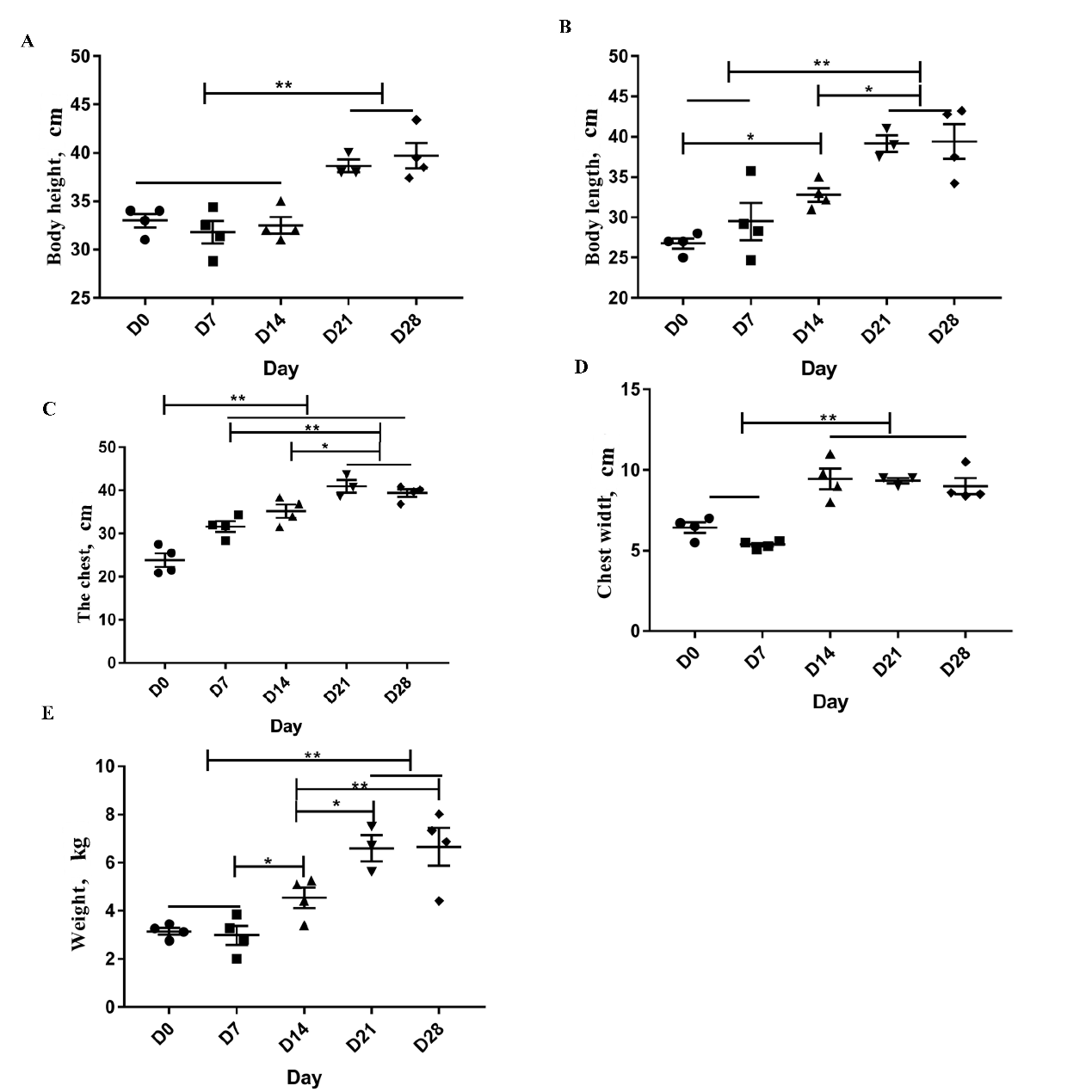


**Fig. S4** Growth performance and rectal temperature testing. **A** Body height. **B** Body length. **C** Chest girth. **D** Chest width. **E** Bodyweight. **F** Rectal temperature. ^*^*P <* 0.05, ^**^*P <* 0.01


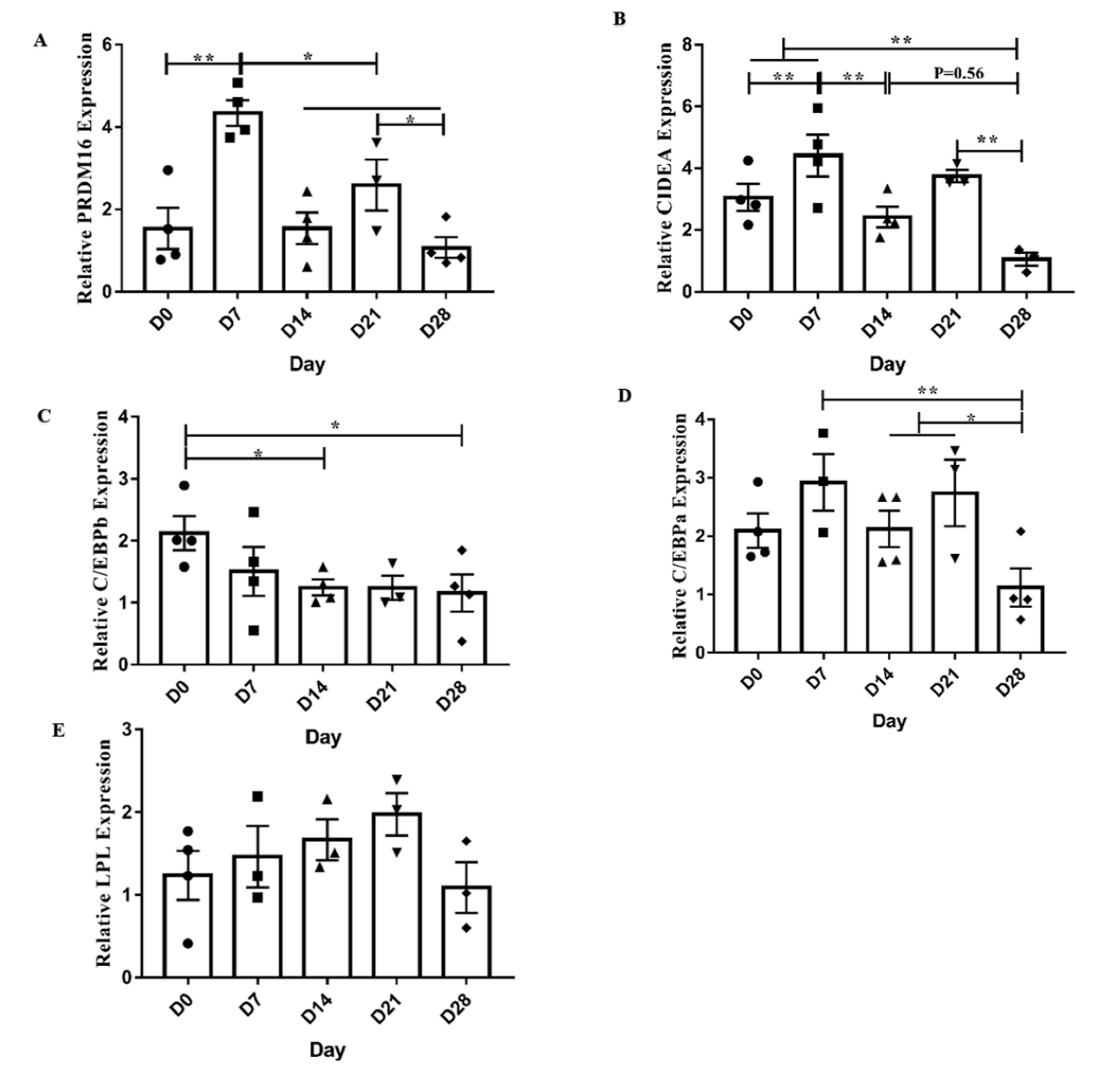


**Fig. S5** Relative gene expression normalized to geometric mean of *PRDM16*, *CIDEA*, *C*/*EBPb*, *C*/*EBPa,* and *LPL.* ^*^*P <* 0.05, ^**^*P <* 0.01
