# Supplementary material for: Global gene expression profiling of perirenal brown adipose tissue whitening in goat kids reveals novel genes linked to adipose remodeling
Source: J Anim Sci Biotechnol. 2024 Mar 14;15:47. doi: 10.1186/s40104-024-00994-w (PMC10938744; doi:10.1186/s40104-024-00994-w)
Supplement: Supplementary file 2 — Additionalfile 2: Table S1. The concentration and purity of total DNA in goat adipose tissue. [file 40104_2024_994_MOESM2_ESM.docx]

**Table S1** The concentration and purity of total DNA in goat adipose tissue

| **Sample** | **Concentration, ng/μL** | **OD_260/280_** |
| --- | --- | --- |
| D0-1 | 288.821 | 1.851 |
| D0-2 | 1,110.561 | 1.838 |
| D0-3 | 1,484.88 | 1.85 |
| D0-4 | 1,458.4 | 1.848 |
| D7-1 | 445.008 | 1.86 |
| D7-2 | 756.241 | 1.867 |
| D7-3 | 658.258 | 1.754 |
| D7-4 | 586.145 | 1.786 |
| D14-1 | 251.635 | 1.893 |
| D14-2 | 151.755 | 1.98 |
| D14-3 | 150.341 | 1.952 |
| D21-1 | 104.288 | 1.885 |
| D21-2 | 758.88 | 1.894 |
| D21-3 | 271.461 | 1.891 |
| D28-1 | 288.201 | 1.795 |
| D28-2 | 297.78 | 1.892 |
| D28-3 | 117.75 | 1.904 |
| D28-4 | 414.831 | 1.857 |
