# Supplementary material for: Global gene expression profiling of perirenal brown adipose tissue whitening in goat kids reveals novel genes linked to adipose remodeling
Source: J Anim Sci Biotechnol. 2024 Mar 14;15:47. doi: 10.1186/s40104-024-00994-w (PMC10938744; doi:10.1186/s40104-024-00994-w)
Supplement: Supplementary file 3 — Additional file 3: Table S2. Primers for qPCR of adipose tissue in goats. [file 40104_2024_994_MOESM3_ESM.docx]

**Table S2** Primers for qPCR of adipose tissue in goats

| **Gene name** | **Primer sequence (5'→3')** | **Product size, bp** |
| --- | --- | --- |
| *UCP1* | F: CAAGATCTCAGCGGGCCTAA | 87 |
|  | R: TGTGCTTGCAGTCTGACCTT |  |
| *DIO2* | F: TATGACTCGGTCATTCTCCTCA | 154 |
|  | R: CACCCAGTTTCACCTGTTTGT |  |
| *UCP2* | F: AGCCAACGGATGTGGTGAA | 101 |
|  | R: TCGGGCAATGGTTTTGTAG |  |
| *PRDM16* | F: GGATAGTGACCGTGACAAGACC | 137 |
|  | R: GGAAGAAGTGCTGGGAGTAGAAG |  |
| *CIDEA* | F: ATTGGCTGCCTCAATGTGAA | 173 |
|  | R: AGCACTCGGAGCATGTAGGT |  |
| *PPARGC1a* | F: TCAGTACAACAACGAGCCTTCA | 119 |
|  | R: GCAATCCGTCTTCATCCACA |  |
| C/*EBPA* | F: ACAAGAACAGCAACGAATACCG | 129 |
|  | R: CATTGTCACTGGTCAGCTCCA |  |
| C/*EBPB* | F: GCACAGCGACGAGTACAAGAT | 188 |
|  | R: TGAACAAGTTCCGCAGGGT |  |
| *PPARG* | F: CGGGAAAGACGACAGACAAA | 148 |
|  | R: AACTGACACCCCTGGAAGATG |  |
| *ADIPOQ* | F: GGGTCACTGTCCCCAATGTT | 175 |
|  | R: TTGTCGTTCTTGTAGAGGCTGA |  |
| *LPL* | F: CTGCCTTATACAAGAGGGAACC | 170 |
|  | R: CACATTGCCCAGGGGATAGT |  |
| mtDNA D-loop | F: CACAAACTTCCCACTCCACAAG | 148 |
|  | R: GTAGGCGAGCGGTGTAATGTAC |  |
| *BAX* | F: TCTCCCCGAGAGGTCTTTTT | 151 |
|  | R: TGATGGTCCTGATCAACTCG |  |
| *BCL2* | F: GAAACCCCGCCACGAATTA | 96 |
|  | R: CCAGCCAACAGTACGGAACAA |  |
| *Caspase*-3 | F: ACAGCACCTGGTTACTTTTCCT | 143 |
|  | R: TCTGTCGCTACCTTTCGGTTA |  |
| *Caspase*-6 | F: CTACTCCGTGGCAGAAGGTTA | 159 |
|  | R: TCTACTCGGCGCTGAGAAAC |  |
| *Caspase*-9 | F: GGCCCTTCCTTTGTTCATCT | 100 |
|  | R: CTCCACATCCTGTTTTGCTG |  |
| *NMRK2* | F: CAGACACCCACATCCTCATTC | 116 |
|  | R: CCTCCACTTGCACTCTTCATAG |  |
| *ACADVL* | F: AGGTGTTTCCATACCCGTCT | 110 |
|  | R: ATTCTTGGCAGCATCGTTC |  |
| *CKB* | F: GAGGTACTATGCGCTCAAAAGC | 153 |
|  | R: TTATCGTTATGCCAGATGCC |  |
| *IDH2* | F: GAGAAGCACTACAAGACCGAGTT | 184 |
|  | R: ACGTCATCAGACCAAGGGAG |  |
| *ACO2* | F: ACATCTCCAACAACCTGCTCA | 128 |
|  | R: GCCGTGTTTCTTGTAGTAGCG |  |
| *CHCHD10* | F: CGCCTATGAGATCAGGCAGTT | 114 |
|  | R: GAGCTCAGACCGTGGTTGTA |  |
| *NDUFV1* | F: CAGAGTCGAGGTGACTGGTACA | 157 |
|  | R: TGCCATCTGAGGGCTTATTC |  |
| *ACAD9* | F: TTCCAGAGGTCAGCCGAGAT | 171 |
|  | R: CTGGGACTTGCATTCCAAAA |  |
| *NDUFA6* | F: CCCAGGGTGGTTGATCTTCT | 116 |
|  | R: CTTGGTGCTTCTGTTTCGTG |  |
| *PTGES2* | F: AGGAAGGTGCCCATCGTTA | 142 |
|  | R: TCATGGGTGGATAGTAGGTGAT |  |
| LBP | F: GGAGCCAGAAAAGATACAAGTG | 189 |
|  | R: ATCTGGAGAATGGGGTCGT |  |
| *ACSF2* | F: GGCACAATGGTGAGCTTGA | 188 |
|  | R: CCTCCACGTATGGTTGAGATG |  |
| *RBP4* | F: GAAAGGAAACGATGACCACTG | 122 |
|  | R: GGGCGAACACGAAAGAGTAG |  |
| *AOX1* | F: GTGATAGCCGATTCTGAGGTC | 163 |
|  | R: ACGCTTCATCCACATTTCC |  |
| *RHOBTB3* | F: CCAGTGGGAAGAGTTAGAAGATG | 152 |
|  | R: GAGGTTTTCTGGCTTGATAGGT |  |
| *HIF1A* | F: TGTCTCCATTACCTGCCTCTG | 182 |
|  | R: TGAACTTTGTCTGGTGCTTCC |  |
| *APCDD1* | F: ACTACTATCACTACTCGGACCCC | 195 |
|  | R: ACTCATTCCCGCTGAAGACG |  |
| *GHR* | F: GTGAGAACCCGACAACGAA | 153 |
|  | R: CACTGTTAGCCCAAGTATTCCA |  |
| *THRSP* | F: CCGATGGAGCTGAGACTGAA | 131 |
|  | R: GGTAAGATGGGTGAGGATGTG |  |
| *EIF4A2* | F: CATGCCAGGGACTTCACAGT | 138 |
|  | R: CACATCAATTCCACGAGCC |  |
| *ANXA1* | F: GCTTGCTGTAAATGACGACTTG | 135 |
|  | R: ACTCTGCGAAGATGGGGAT |  |
| *ZFAND5* | F: CCCAAACCAAAGAAGAACAGG | 104 |
|  | R: GAGTAACGGTGAAGTCCACAAA |  |
| DNA β-Actin | F: CAGCAGATGTGGATCAGCAAG | 89 |
|  | R: CGCAACTAACAGTCCGCCTA |  |
| *ACTIN* | F: CCTGCGGCATTCACGAAACTAC | 87 |
|  | R: ACAGCACCGTGTTGGCGTAGAG |  |
