# Supplementary material for: Global gene expression profiling of perirenal brown adipose tissue whitening in goat kids reveals novel genes linked to adipose remodeling
Source: J Anim Sci Biotechnol. 2024 Mar 14;15:47. doi: 10.1186/s40104-024-00994-w (PMC10938744; doi:10.1186/s40104-024-00994-w)
Supplement: Supplementary file 4 — Additional file 4: Table S3. Pre-weaning kid mortality statistics (2011–2018). [file 40104_2024_994_MOESM4_ESM.docx]

**Table S3** Pre-weaning kid mortality statistics (2011–2018)

| **Item** | **Mortality** | **Survival** |
| --- | --- | --- |
| Four seasons | 436 | 245 |
| Below average birth weight (2.17 kg) | 234 | 43 |
| Above average birth weight (2.17 kg) | 202 | 202 |
| Winter | 134 | 53 |
| Below average birth weight (2.17 kg) | 75 | 32 |
| Above average birth weight (2.17 kg) | 59 | 21 |
