# Supplementary figures and images for: Global gene expression profiling of perirenal brown adipose tissue whitening in goat kids reveals novel genes linked to adipose remodeling
Source: J Anim Sci Biotechnol. 2024 Mar 14;15:47. doi: 10.1186/s40104-024-00994-w (PMC10938744; doi:10.1186/s40104-024-00994-w)

**Table S4** Overview of sequencing data


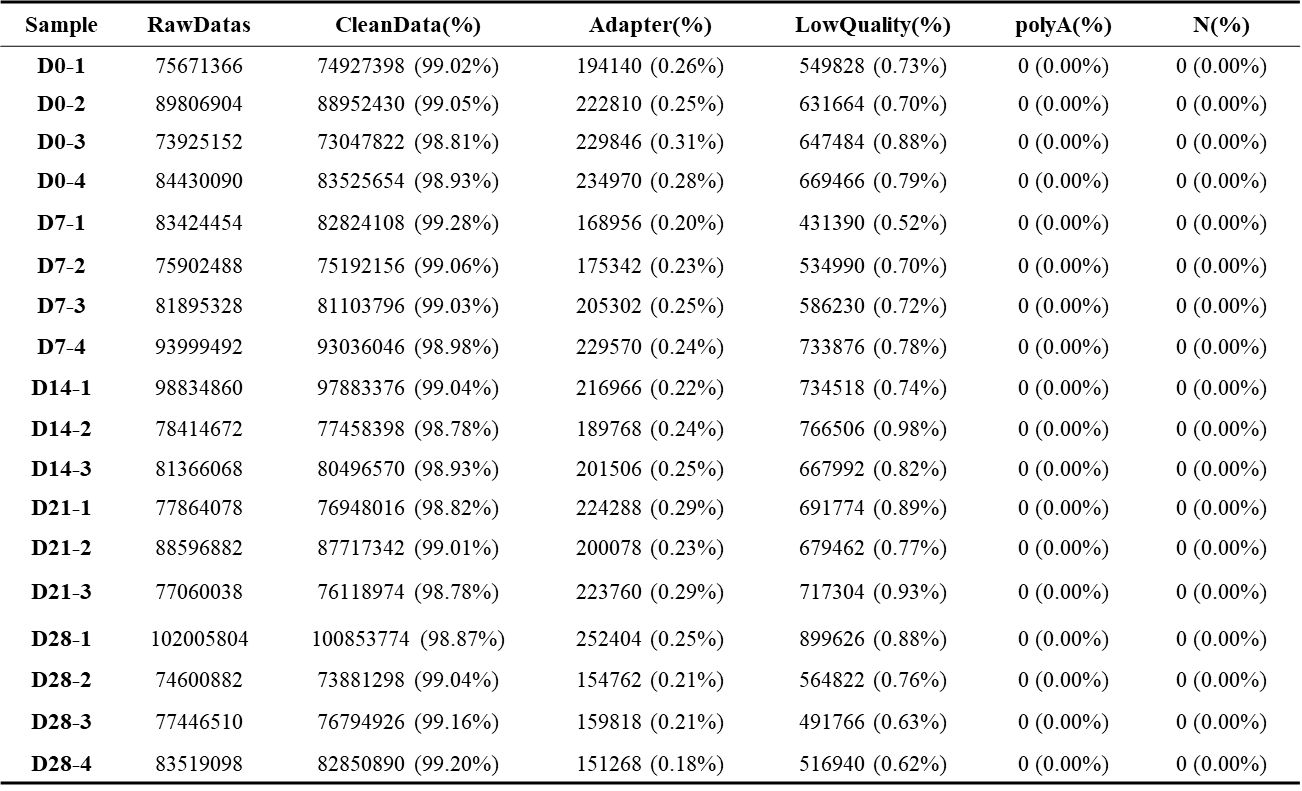

Supplement: Supplementary file 5 — Additional file 5: Table S4. Overview of Sequencing Data. [file 40104_2024_994_MOESM5_ESM.docx]

**Table S5** Bases mass analysis


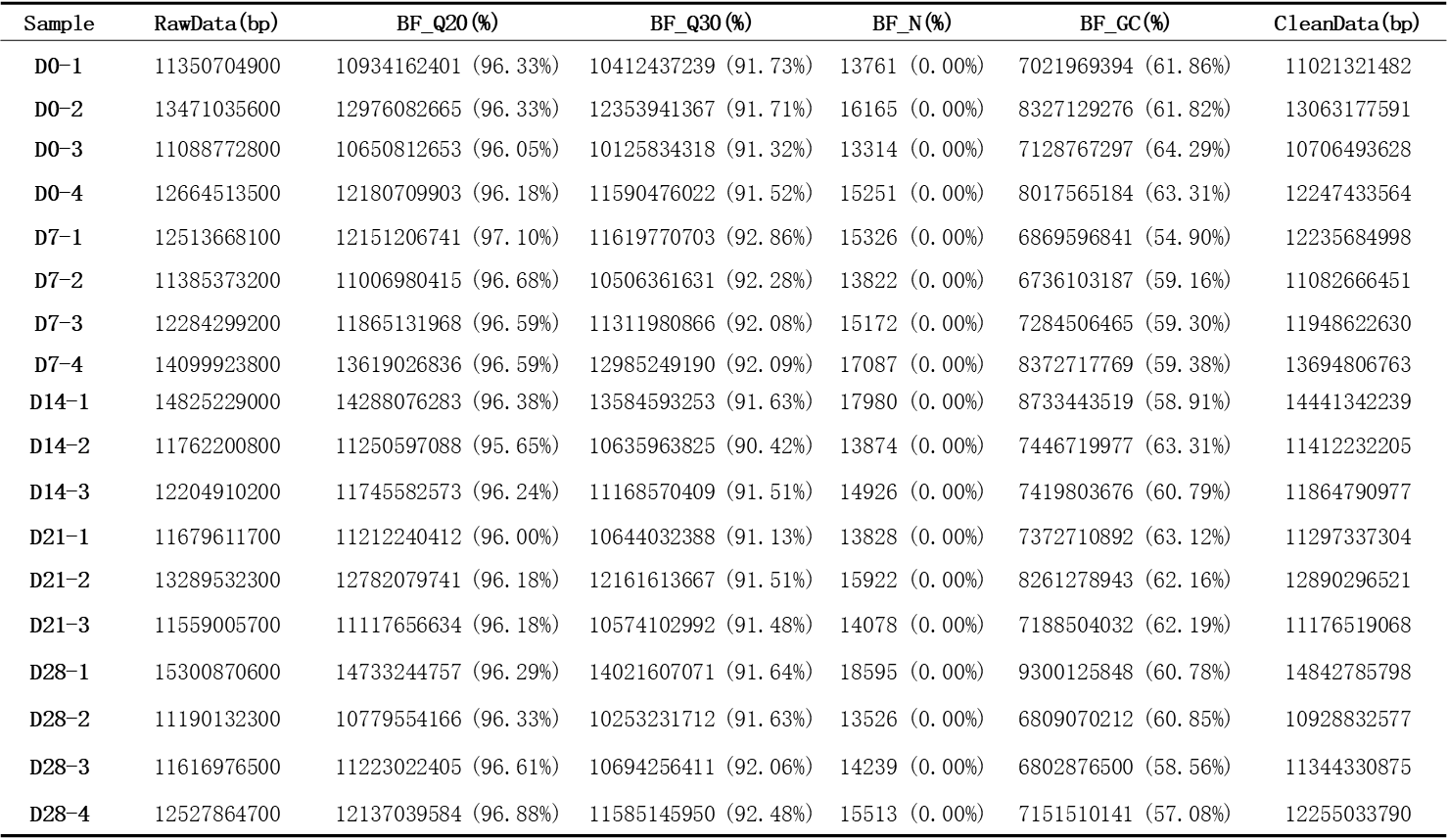


*bp* Base pair

Supplement: Supplementary file 6 — Additional file 6: Table S5. Bases mass analysis. [file 40104_2024_994_MOESM6_ESM.docx]

**Table S6** Reference genome alignment


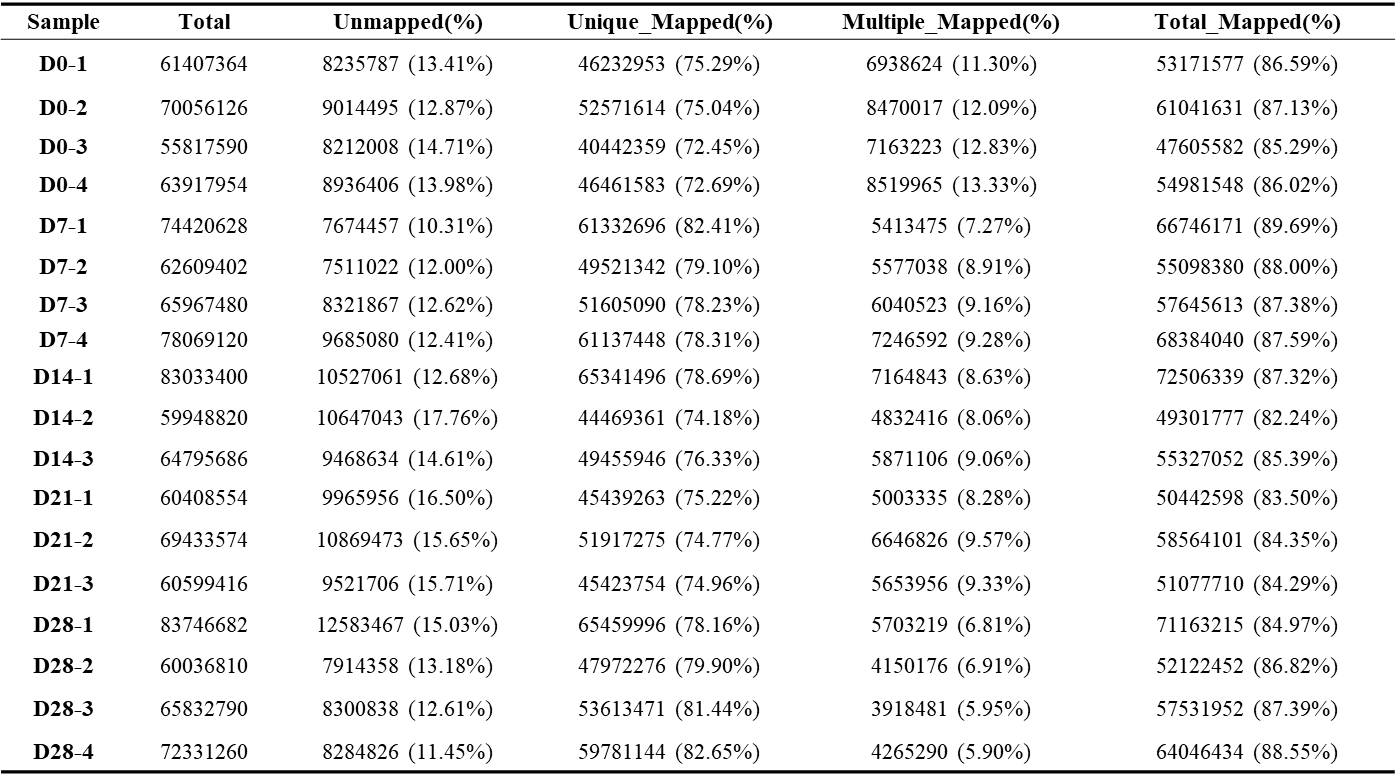

Supplement: Supplementary file 7 — Additional file 7: Table S6. Reference genome alignment. [file 40104_2024_994_MOESM7_ESM.docx]

**Table S7** Comparison of reference area


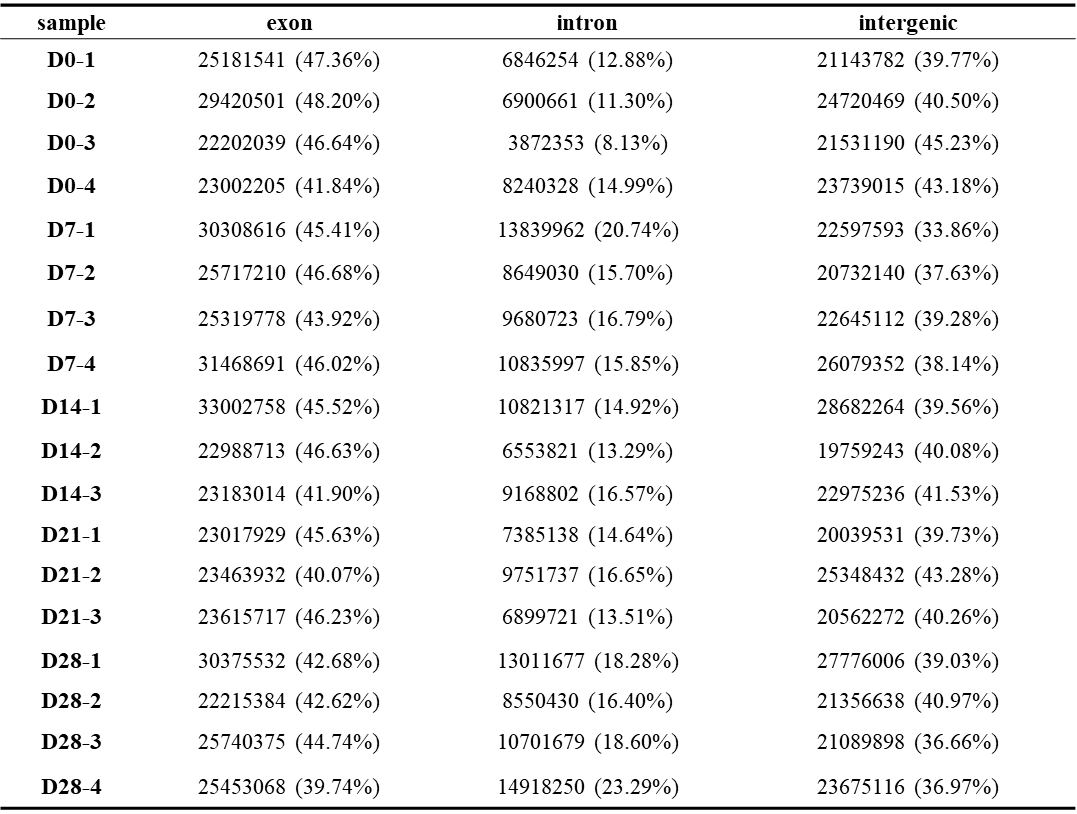

Supplement: Supplementary file 8 — Additional file 8: Table S7. Comparison of reference area. [file 40104_2024_994_MOESM8_ESM.docx]
